# Chemical composition and pharmacological mechanism of ephedra-glycyrrhiza drug pair against coronavirus disease 2019 (COVID-19)

**DOI:** 10.18632/aging.202622

**Published:** 2021-02-13

**Authors:** Xiaoling Li, Qin Qiu, Mingyue Li, Haowen Lin, Shilin Cao, Qu Wang, Zishi Chen, Wenhao Jiang, Wen Zhang, Yuge Huang, Hui Luo, Lianxiang Luo

**Affiliations:** 1 Animal Experiment Center of Guangdong Medical University, Zhanjiang 524023, Guangdong, China; 2 Graduate School of Guangdong Medical University, Zhanjiang 524023, Guangdong, China; 3 Department of Pathology and Laboratory Medicine, Perelman School of Medicine, University of Pennsylvania, Philadelphia, PA 19104, USA; 4 The First Clinical College of Guangdong Medical University, Zhanjiang 524023, Guangdong, China; 5 Group of Sustainable Biochemical Engineering, School of Food Science and Engineering, Foshan University, Foshan 528000, Guangdong, China; 6 Sustainable Biochemical and Biosynthetic Engineering Center, Foshan Wu-Yuan Biotechnology Co., Ltd., Guangdong Biomedical Industrial Base, Foshan 528000, Guangdong, China; 7 Aditegen LLC, Jersey, NJ 07310, USA; 8 Department of Pediatrics, the Affiliated Hospital of Guangdong Medical University, Zhanjiang 524001, Guangdong, China; 9 The Marine Biomedical Research Institute, Guangdong Medical University, Zhanjiang 524023, Guangdong, China; 10 Marine Medical Research Institute of Zhanjiang, Zhanjiang 524023, Guangdong, China

**Keywords:** COVID-19, ephedra, glycyrrhiza, network pharmacology, molecular docking

## Abstract

Traditional Chinese medicine (TCM) had demonstrated effectiveness in the prevention and control of COVID-19. Statistics showed that Ephedra and Glycyrrhiza were frequently used in the treatment of COVID-19. We hypothesized that the Ephedra-Glycyrrhiza drug pair is a potential choice for the treatment of COVID-19. Here, 112 active compounds were identified from Ephedra-Glycyrrhiza via network pharmacology approach. Ephedra-Glycyrrhiza pair enrichment analysis demonstrated that these compounds might participate in the cAMP, PI3K-Akt, JAK-STAT and chemokine signaling pathways, which had a high correlation with respiratory, nervous, blood circulation and digestive system-related diseases. Pathway analysis between Ephedra-Glycyrrhiza and COVID-19 showed that the key targets were TNF-α, IL2, FOS, ALB, and PTGS2. They might control PI3K-Akt signaling pathway to exert immune regulation, organ protection and antiviral effects. Molecular docking results showed that the active compounds from the Ephedra-Glycyrrhiza pair bound well to COVID-19 related targets, including the main protease (Mpro, also called 3CLpro), the spike protein (S protein), and the angiotensin-converting enzyme 2 (ACE2). The Molecular dynamics simulation was analyzed for the stability and flexibility of the complex. In conclusion, our study elucidated the potential pharmacological mechanism of Ephedra-Glycyrrhiza in the treatment of COVID-19 through multiple targets and pathways.

## INTRODUCTION

The coronavirus disease 2019 (COVID-19) pandemic is caused by severe acute respiratory syndrome coronavirus 2 (SARS-CoV-2), and has had a huge negative impact on both mental and physical health, and on economic development as well. [1]. The number of COVID-19 cases is still continuously growing. As of Nov 18^th^, 2020, more than 56 million cases of COVID-19 have been confirmed with more than 1 million deaths worldwide (according to data from Johns Hopkins University). The most common symptoms of COVID-19 patients are fever, fatigue, headache, cough, diarrhoea, sore throat and other multiple organ system symptoms. Severe patients rapidly develop into acute respiratory distress syndrome (ARDS), multiple organ dysfunction syndrome (MODS) and even death [2]. Accumulated evidence has suggested that patients with severe COVID-19 suffered from cytokine storm syndrome, which involved a large number of cytokines such as IL-1, IL-6 and interferon-γ circulating in the blood, tissues and organs through a positive feedback loop between cytokines and immune cells, eventually triggering ARDS and MODS, as well as death [3, 4]. A pneumonia outbreak associated with a new coronavirus of probable bat origin.

SARS-CoV-2 along with the severe acute respiratory syndrome coronavirus (SARS-CoV) and the Middle Eastern respiratory syndrome-related coronavirus (MERS-CoV) are the top three life-threatening coronaviruses that affect humans [5, 6]. The pathogenic mechanism of coronavirus includes four steps: attachment/entry, replication/transcription, mature virus assembly and release [7]. SARS-CoV-2 is an enveloped, positive-sense, single-stranded RNA virus, which encodes four structural proteins: (spike (s), envelope (e), membrane (m) and nucleocapsid (n)) [8]. Spike protein (S protein) promotes host infection and virus-cell membrane fusion through spontaneously conformation changes [9]. SARS-CoV-2 uses the angiotensin converting enzyme II (ACE2) to gain entry into cells, which is also the receptor of SARS-CoV entrance [5, 10]. Notably, ACE2 binding to S protein is the necessary prerequisite to the entrance of SARS-CoV-2 into host cells, so ACE2 has been considered to be the "doorknob" for SARS-CoV-2 infection [11]. Furthermore, SARS-CoV-2 replication and transcription depend on several non-structural proteins such as main protease (Mpro), RNA-dependent RNA-polymerase (RdRp) and papain-like protease (PLpro) [12]. Lopinavir and ritonavir that block the cleavage function of Mpro were expected to inhibit virus replication [8, 13]. But the efficacy of lopinavir and ritonavir are not fully explored, so there are no specific drugs against SARS-CoV-2.

TCM has been approved to exert therapeutic or preventive roles in infectious diseases for thousands of years [14, 15]. When summarizing the experience of using traditional Chinese herbs to prevent and treat COVID-19 in TCM, we found that Ephedra and Glycyrrhiza were frequently and widely used [16, 17]. Glycyrrhiza has antiviral, anti-inflammatory, antifungal, anticancer, antioxidant, and cytotoxic effects, whose most important component is glycyrrhizin [18, 19]. Many studies have shown glycyrrhiza is an effective therapeutic for the treatment of SARS-CoV [20, 21]. In addition, glycyrrhizin inhibits SARS-CoV viral adsorption and penetration effectively *in vitro* [22, 23]. Ephedra showed antiviral, anti-inflammatory, antibacterial, antioxidant, diuretic activities, etc [24]. It is widely used in respiratory diseases such as asthma, influenza and colds, and could release a range of symptoms including fever, headache, nasal congestion and cough [25]. The compatibility of Ephedra and Glycyrrhiza are complementary, mutually promoting and mutually restricting. Mutual supplement and promotion as the different action, restricting each other as the opposite function, thereby bringing their own advantages into full play in the compatibility and enhancing the therapeutic effect. The Ephedra-Glycyrrhiza drug pair (EG) was well recorded in Zhang Zhongjing in the Golden Chamber Synopsis, has been widely used for the treatment of colds and bronchial asthma in the clinic [26]. Both Glycyrrhiza and Ephedra have shown anti-inflammatory, lung injury and immunomodulatory properties [27–30]. However, there are no relevant research reports on the mechanism of EG pair for the treatment of COVID-19.

Due to the limitation of experimental conditions and to increase research efficacy, computer network technology is an efficient approach to predict effective therapeutic against COVID-19 in a low-cost way. Network pharmacology is a new discipline that designs drugs based on the theory of biology. The biological system network analysis reveals the mystery of multi-molecular drugs in the cooperative treatment against diseases through multi-components, multi-targets, multi-pathways [31]. It is similar to the research of TCM syndrome, emphasizing a comprehensive understanding of the aetiology and mechanisms. Molecular docking predicts binding poses and binding affinities through the interactions between receptors and drug molecular ligands, which involves spatial matching and energy matching between molecules [32]. Molecular dynamics simulates various movements of atoms and molecules in a defined system in a certain period of time through Newtonian mechanics, and then evaluates the stability and flexibility of the system [33]. These methods are powerful tools for pharmacological mechanistic research, drug research and development [34, 35]. Therefore, in this study, network pharmacology, molecular docking and molecular dynamics were used to explore the pharmacological targets and mechanisms of EG against COVID-19.

## RESULTS

### Active compounds analysis

After the ADME screening, 23 active compounds from Ephedra (Supplementary Table 1) and 92 active compounds from Glycyrrhiza (Supplementary Table 2) were obtained in TCMSP. Among the 23 compounds of Ephedra, the flavonoids eriodictyol, naringenin and leucopelargonidin (OB ≥ 50%) have anti-inflammation, immune-regulation and antioxidant stress effects [36–38]. In Glycyrrhiza, 42 compounds were with OB ≥ 50%. For example, glycyro (OB =90.78%) has several biological activities such as antioxidant, anti-inflammatory, antibacterial, anti-angiogenesis and anti-allergy [39], which could treat arthritis by regulating autoimmunity and inflammation [40]; licopyranocoumarin (OB =80.36%) is effective against HIV infection by acting on the adsorption and invasion process of HIV [41]; naringenin (OB =59.29%) has therapeutic effects on oxidative stress, inflammation, cancer, diabetes, cardiovascular disease and nervous system disease [42]. Notably, quercetin (OB =46.43%) is an active compound of both Ephedra and Glycyrrhiza, which has several useful pharmacological activities such as anti-inflammatory, immunomodulatory [43, 44] and antiviral [45]. These components of EG have great possibilities as the key components for treating COVID-19.

Traditional Chinese medicine pays attention to channel tropism, which refers to the close relationship between drug action and human meridians and collaterals, showing the selectivity of a drug action on a certain part of the human body. From ETCM, Ephedra belongs to the lung and bladder meridian, while glycyrrhiza belongs to the lung, spleen, stomach and heart meridian, demonstrating Ephedra can work in lung, spleen, stomach and heart. The details are described in Supplementary Table 3.

### Drug targets and COVID-19 targets analysis

**Figure 1:**
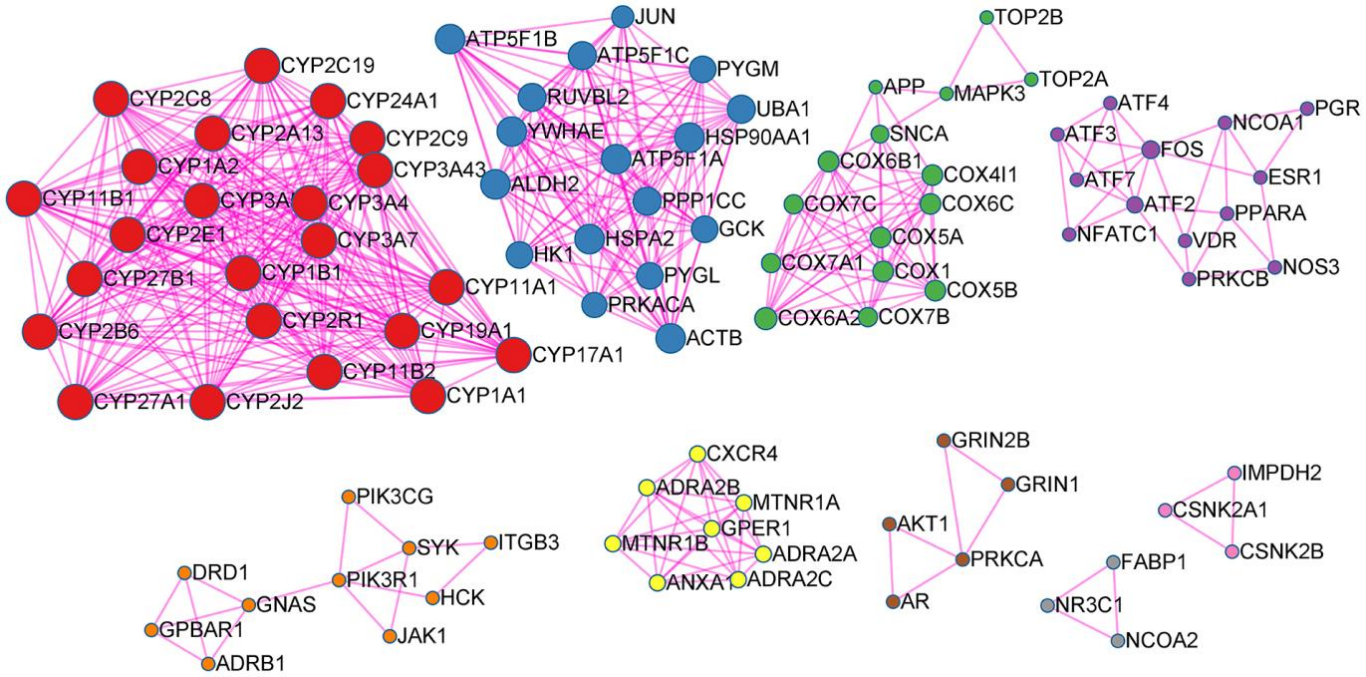
**EG PPI network.** Points represent targets, lines represent interaction relationships and different colors represent different clusters.

The 339 targets of glycyrrhiza and 122 targets of Ephedra were obtained from the ETCM database, and 71 targets belonged to both Ephedra and Glycyrrhiza. The 226 human genes of novel corona pneumonia were issued by Genecards. MCODE plug-in was used to confirm the PPI network function clusters. The original parameter was also used to construct a list of the corresponding meaningful clusters presented (Figure 1). Cluster 1 (score: 11) consisted of 23 nodes, and its seed gene was CYP2R1. It has D-25-hydroxylase activity on both vitamin D, and CYP2R1 deficiency can cause rickets vitamin D-dependent type 1B (VDDR1B) [46]. Cluster 2 (score: 7.41) consisted of 17 nodes and its seed gene was ATP5F1C. In the presence of a transmembrane proton gradient, mitochondrial membrane ATP synthetase (ATP synthetase or complex V) produces ATP from ADP as part of the electron transport complex of the respiratory chain. Cluster 3 (score: 3.93) consisted of 15 nodes, and its seed gene was MAPK3. MAPK3, a member of the MAP kinase family involves in signalling cascades that regulate various cellular processes, such as proliferation, differentiation and cell cycle progression, in response to various extracellular signals [47]. Cluster 4 (score 2.15) consisted of 13 nodes, and the seed gene was ESR1. Oestrogen receptor (ER) and NF-kappa-B inhibit each other in specific cell types, reducing NF-kappa-B DNA binding activity, inhibiting NF-kappa-B-mediated transcription of IL6 promoter, and replacing rela/p65 and promoter-related co-regulators [48]. Cluster 5 (score 3.5) consisted of 8 nodes, and the seed gene was ADRA2B. ADRA2B, Alpha-2B adrenergic receptor, mediates the catecholamine-induced inhibition of adenylate cyclase through the action of G proteins [49]. Cluster 6 (score 1.5) consisted of 10 nodes, and the seed gene was SYK. Cluster 7 (score 1.2) consisted of 5 nodes, and the seed gene was GRIN1. Cluster 8 and Cluster 9 (score 1) consisted of 3 nodes with CSNK2A1 and NR as the seed genes, respectively.

### GO and pathway enrichment analysis of EG

**Figure 2:**
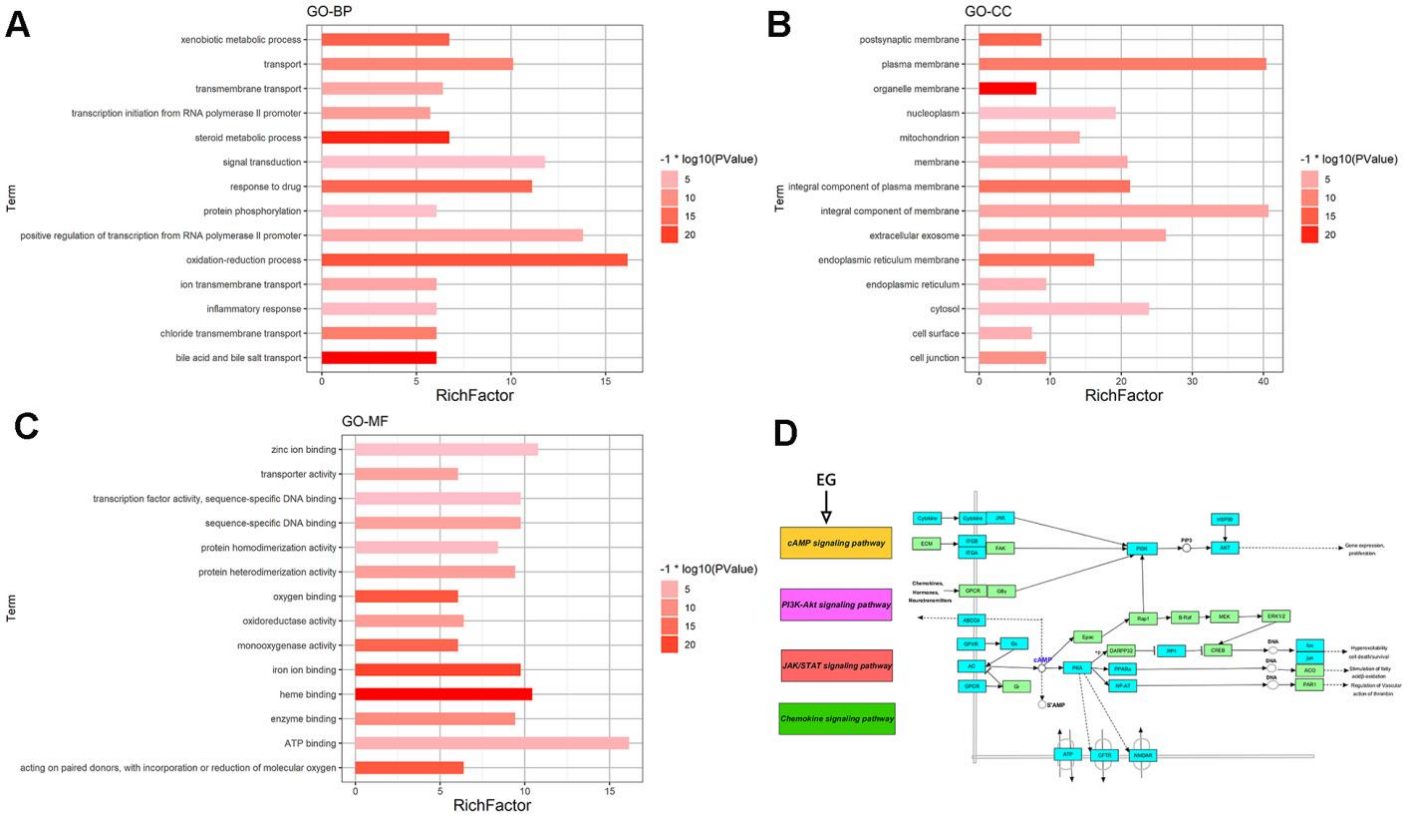
**GO and pathway enrichment analysis of EG.** (**A**) The top 15 biological processes. (**B**) The top 15 cellular components. (**C**) The top 15 molecular functions. The colorful scales indicate the different thresholds for the p-values, and the lengths of the lines represent the number of genes corresponding to each term. (**D**) The cAMP signalling pathway. The large purple, red, and green rectangles on the left represent the key terms involved in the cAMP signalling pathway. The cAMP signalling pathway itself is in orange. The small green rectangles on the right represent the targets of the pathways themselves, and the blue represent the targets involved in these pathways in EG.

This analysis can identify GO Terms that are statistically significantly enriched. For the GO-BP enrichment analysis, the top 15 significantly enriched GO-BP terms (p<0.05) were listed in Figure 2A. The results distinctly demonstrated that numerous targets involved in various biological processes associated with metabolic process, signal transduction and inflammation, oxidation-reduction process, positive regulation of transcription from RNA polymerase II promoter, drug response, etc. The GO-CC enrichment analysis results were listed in Figure 2B. They showed that EG mainly acted on the cell membrane, plasma membrane and extracellular space. For the GO-MF enrichment analysis, its results were listed in Figure 2C. The results demonstrated that targets of EG were involved in ATP binding, zinc ion binding, haem binding, iron ion binding, and sequence-specific DNA binding. The cAMP signalling pathway was mainly related to the PI3K-Akt, JAK/STAT and the chemokine signalling pathways (Figure 2D).

### Relevant disease-target network analysis

**Figure 3:**
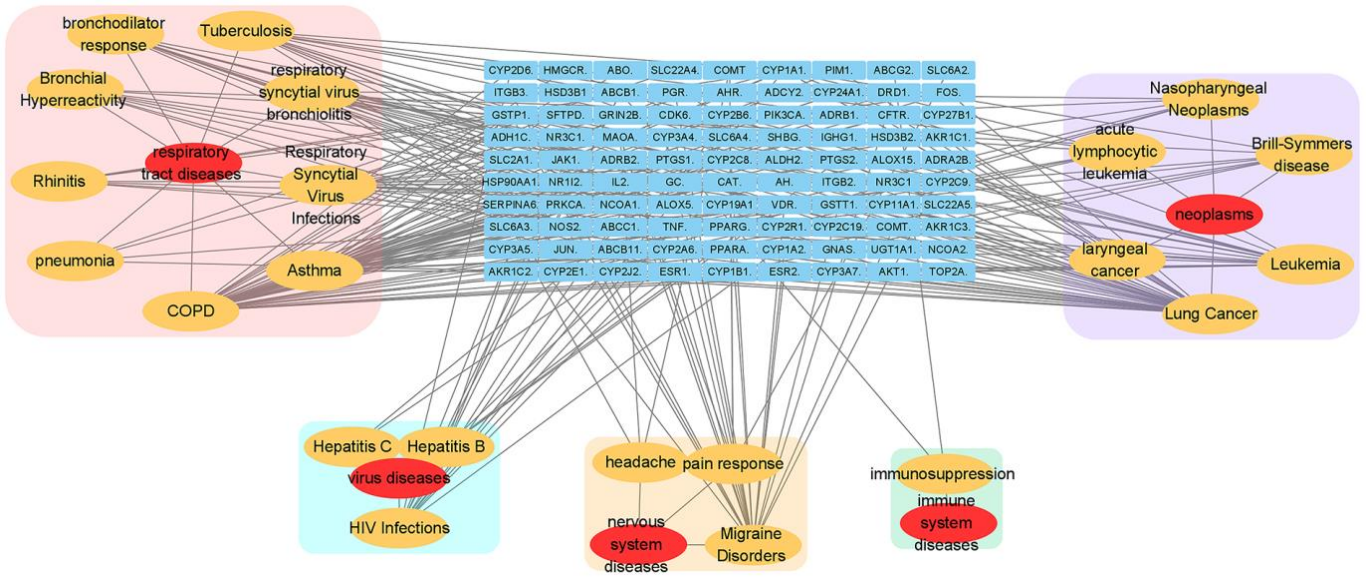
**Relevant disease-target network of EG.** The blue rectangles represent the targets of EG. The yellow ellipses are the related pathways and the red ones are their profile. Each category is filled with rounded rectangles of different colors. The pink one is respiratory tract disease. The peppermint green one is virus disease. The yellow one is nervous system disease. The green one belongs to immune system disease.

Analysis of the relevant disease-target network (Figure 3) demonstrated that these targets had high-degree correlations with 1) respiratory tract diseases: chronic obstructive pulmonary diseases, asthma, respiratory syncytial virus infections, tuberculosis, respiratory syncytial virus bronchiolitis, bronchodilator response, bronchial hyper-reactivity, rhinitis, pneumonia; 2) neoplasms: lung cancer, leukaemia, Brill-Symmers disease, acute lymphocytic leukaemia, nasopharyngeal neoplasms, laryngeal cancer; 3) other virus diseases other than respiratory infection: HIV infections, hepatitis b and hepatitis c infections; 4) nervous system diseases: migraine disorders, pain response, headache; 5) immune system diseases: immunosuppression.

### Mapping relation analysis

**Figure 4:**
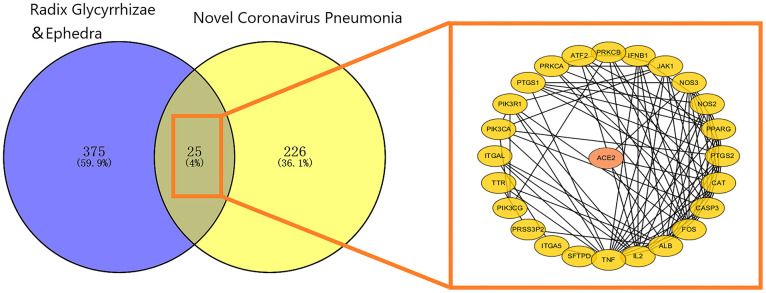
**Mapping relation between Glycyrrhiza and Ephedra and COVID-19.** The 25 intersected targets are presented as a PPI network. The ellipse represents the target, and the black line represents the relationship between the targets.

**Figure 5:**
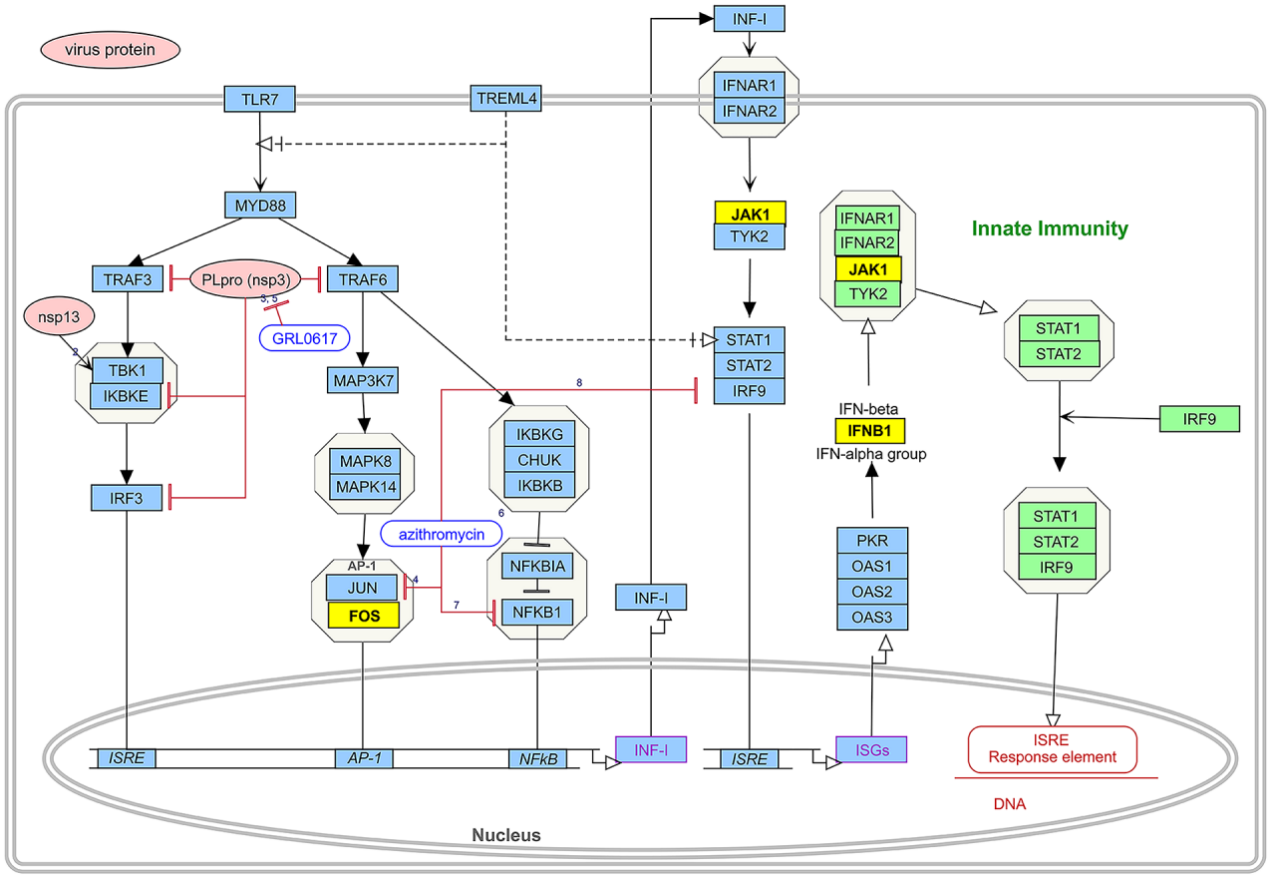
**The induction of type I interferons and signalling leading to the innate immune response during SARS-CoV-2 infection and drug pair action site.** The route consists of two parts. The pink boxes represent virus proteins and the blue boxes represent the targets of type I interference induction and signalling during SARS-CoV-2 infection. Green boxes represent part of the pathway of type I interferon-mediated innate immunity. Yellow boxes represent intersection targets between EG and novel coronavirus pneumonia. Different forms of lines represent interactions between targets.

Twenty-five mapping targets and their relationship networks were obtained by mapping relationship analysis (Figure 4) with the summarized data in Supplementary Table 3. Among them, TNF, FOS, IL2, ALB, and CASP3 rank ahead in degree in the network. In addition, studies have shown that SARS-CoV-2 could trigger the innate immune response via the induction of type I interferons and signalling during infection, and FOS, JAK1, and IFNB1 are involved in this signalling process, as detailed in Figure 5.

### Enrichment analysis of mapped targets (EG anti-COVID-19 targets)

**Figure 6:**
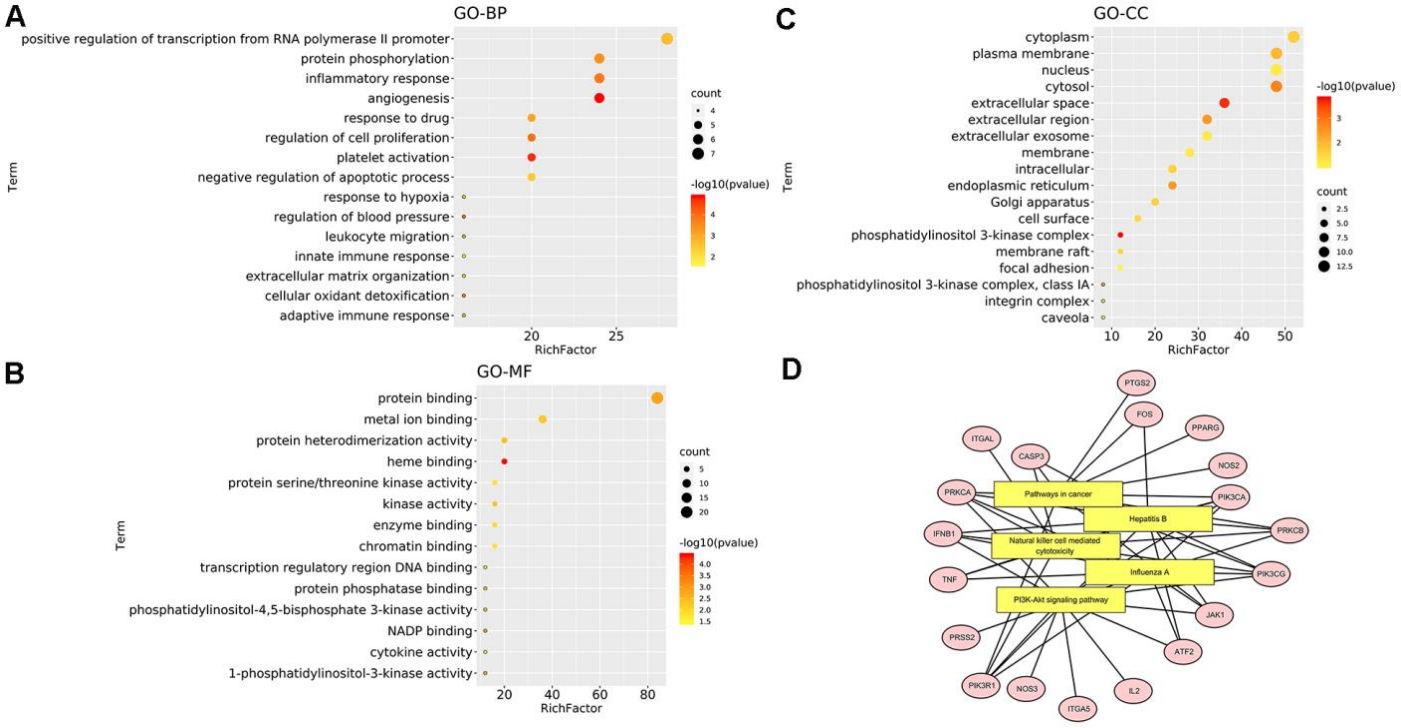
**Enrichment analysis of mapped targets (EG anti-COVID-19 targets).** (**A**) The top 15 biological processes. (**B**) The top 15 cellular components. (**C**) The top 15 molecular functions. The colour scales indicate the different thresholds for the p-values, and the sizes of the dots represent the number of genes corresponding to each term. (**D**) The top 5 KEGG pathways. The yellow rectangles represent KEGG terms while the pink ellipses represent targets involved in pathways. The black line reflects how the target participates in the pathway.

From the GO analysis, these targets involved in angiogenesis, positive regulation of transcription from RNA polymerase II promoter, inflammatory response, protein phosphorylation and other biological processes (Figure 6A). Cell components (CC) were mainly enriched in cell membrane, plasma membrane and extracellular space (Figure 6C). Additionally, from GO molecular function (GOMF), these targets involved in protein binding, metal ion binding, haem binding, protein heterodimerization, kinase activation and other molecular functions (Figure 6B). The top 5 KEGG pathways were visualized in Figure 6D. EG anti-COVID-19 targets were mainly enriched in the PI3K-Akt signalling pathway.

### Compound-virus network construction

**Figure 7:**
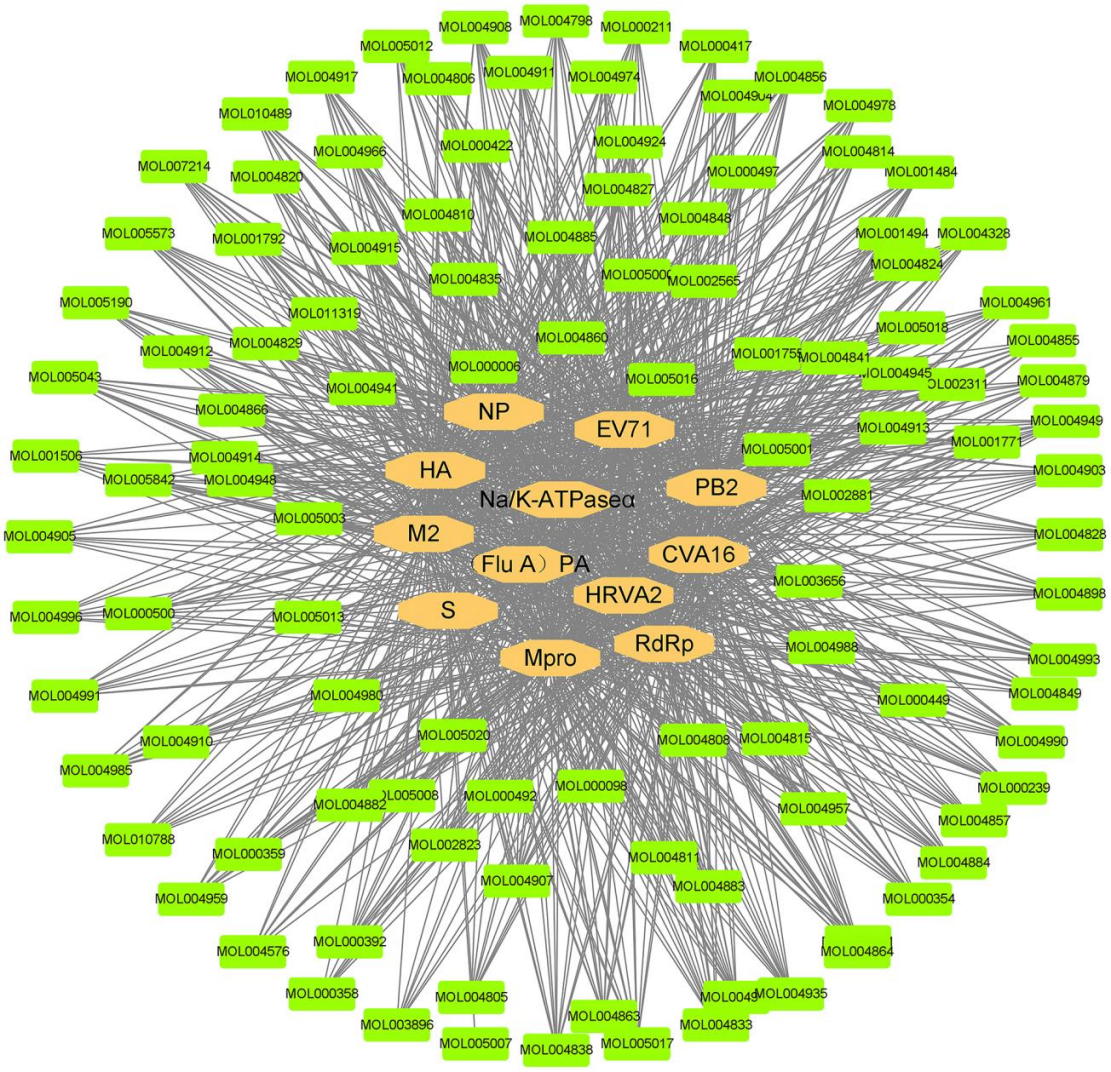
**Compound and virus infection target network.** The orange ellipses are the targets of the novel coronavirus. The green nodes represent the active compounds that can act on the targets of the novel coronavirus. The black line represents the relationship between the targets and the compounds.

The compound-relative targets network consisted of 12 virus protein targets together with 112 active compounds and was presented in Figure 7. Then, the Cytoscape network analyser plug-in was used for topology analysis. The following compounds: dehydroglyasperins C (MOL005020), phaseol (MOL005017), gancaonin H (MOL005001), 7,2',4'-trihydroxy-5-methoxy-3-arylcoumarin (MOL004990), 6-prenylated eriodictyo (MOL004989), kanzonol F (MOL004988), inflacoumarin A (MOL004980), isolicoflavonol (MOL004949), sigmoidin-B (MOL004935), (-)-medicocarpin (MOL004924), 5-prenylbutein (MOL004898), shinpterocarpin (MOL004891), licoisoflavone B (MOL004884), licocoumarone (MOL004882), gancaonin L (MOL004863), kanzonol U (MOL004838), phaseolinisoflavan (MOL004833), glyasperin F (MOL004810), kaempferol (MOL000422), and quercetin (MOL000098) had the degree value of 12, meaning that 20 (20/112) active compounds act on 12 virus targets, indicating that most of the compounds regulated multiple targets to exert various therapeutic effects.

**Figure 8:**
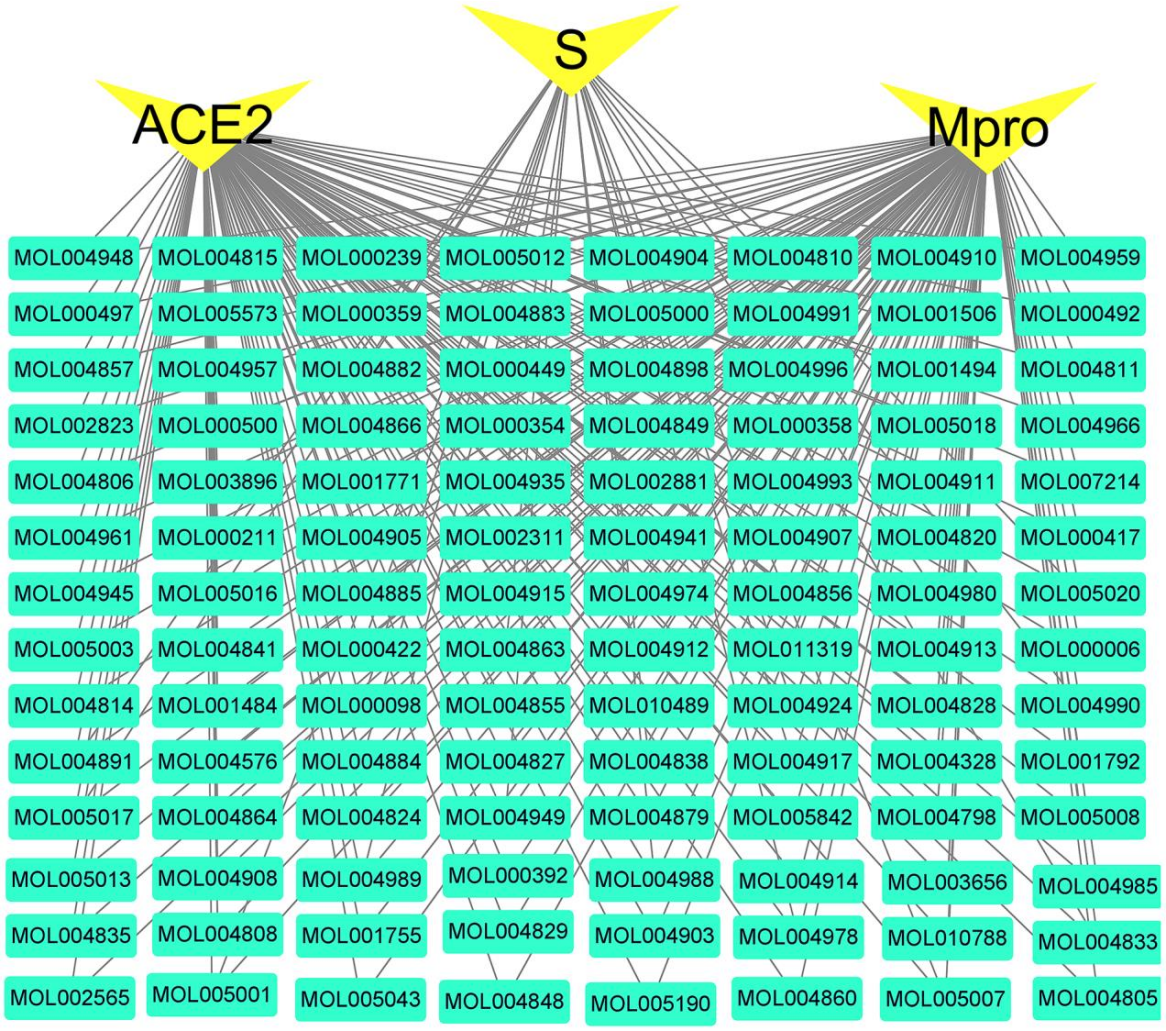
**Active compounds-COVID-19 targets network.** The green nodes are active compounds. The yellow nodes are key targets of the novel coronavirus. The black line represents the relationship between the targets and the compound.

The network between the three viral indicators (ACE2, Mpro, S protein) closely related to COVID-19 and the 112 active compounds are shown in Figure 8. Among them, 112 (112/112) active compounds acted on Mpro, 110 (110/112) could bind to ACE2, and 24 could bind to S protein.

### Molecular docking

The docking results of the 112 active compounds with Mpro, ACE2 and S protein were shown in Supplementary Table 4. Compound gancaonin H showed the lowest affinities but the highest binding energy with Mpro (−19.7 kJ/mol); xambioona was found to have the highest affinity but the lowest binding energy with ACE2 (−50.5 kJ/mol); licorice glycoside E had highest affinity but the lowest binding energy with Mpro (−40.65 kJ/mol) and S protein (−36.10 kJ/mol). The analysis of the binding energy showed that the active compounds formed stable conformations with the three targets. The docking results of xambioona and licorice glycoside E were compared with the positive control compounds as shown in Supplementary Table 5. In terms of binding energy, the affinity of licorice glycoside E was better than that of baicalin, lopinavir, and arbidol which are positive controls for Mpro and S protein, and xambioona had better binding than them against ACE2. The results indicated that the EG-active compounds have good binding activities with Mpro, ACE2 and S protein.

**Figure 9:**
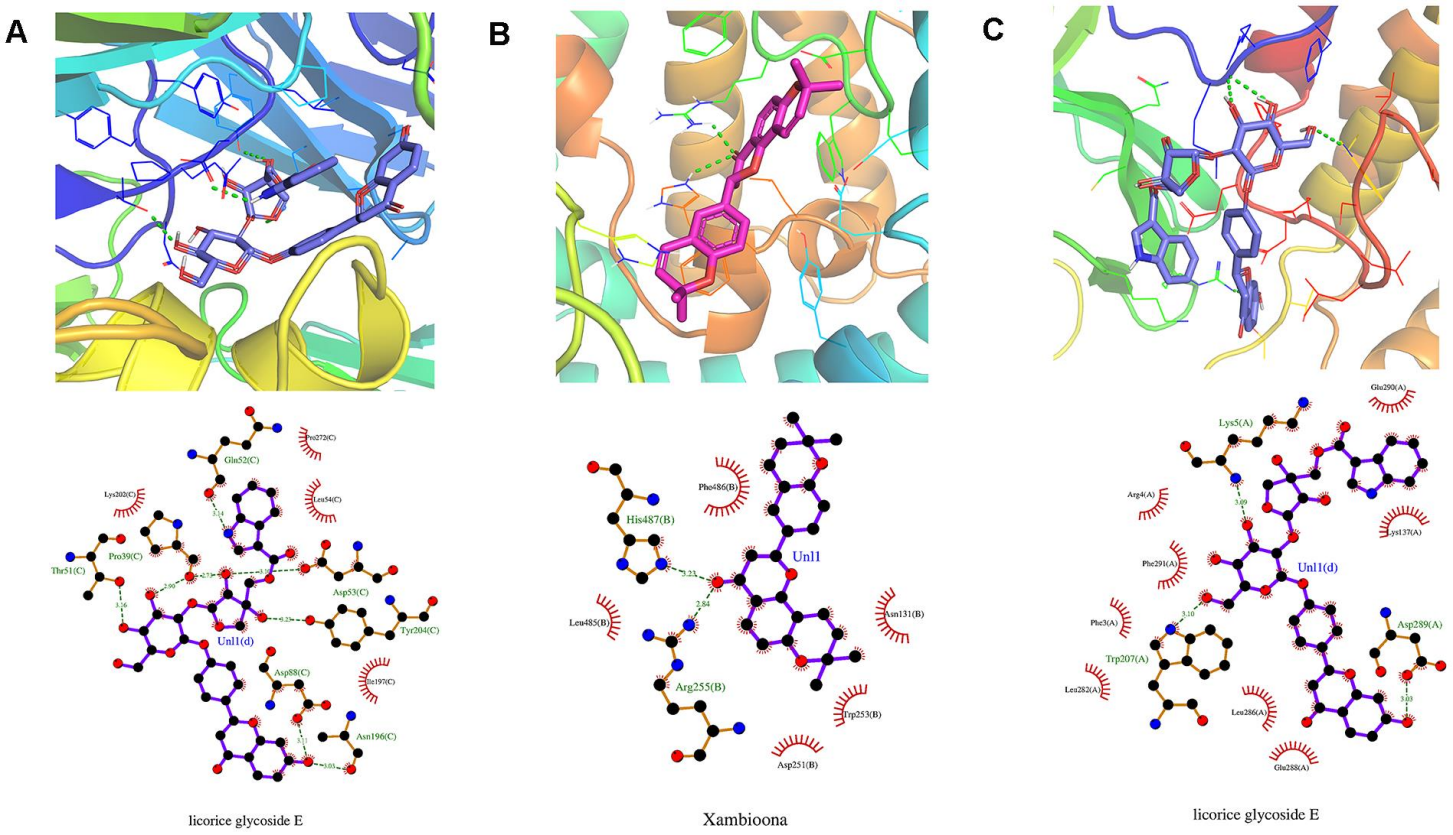
**Molecular models of the selected compounds binding to the target proteins.** (**A**) The docking mode and interactions between xambioona and ACE2, (**B**) Licorice glycoside E and S protein, (**C**) Licorice glycoside E and Mpro. The light red stick represents the binding energy of xambioona, the light blue stick represents the binding energy of licorice glycoside E, and the green dotted line represents the hydrogen bond.

Additionally, the docking analysis between the selected compounds and the target proteins was shown in Figure 9. Compound xambioona bounded to ACE2, forming hydrogen bond interactions with residues His487 and Arg255, and had hydrophobic interactions with Asp251, Phe486, Asn131, Leu485 and Trp253 (Figure 9A). When binding to S protein, licorice glycoside E formed hydrogen bond interactions with residues Pro39, Asp88, Asn196, Tyr204, Asp53, and Gln52; formed hydrophobic interactions with Lys202, Pro272, Leu54, and Tle197 (Figure 9B). In addition, when binding to Mpro, licorice glycoside E formed hydrogen bond interactions with residues Glu166, Gln189, and Thr190; formed hydrophobic interactions with His164, Gln192, Met165, Arg188, and His41 (Figure 9C). Thus, EG binded to target proteins primarily through hydrogen bond and hydrophobic interactions.

### Molecular dynamics simulation

**Figure 10:**
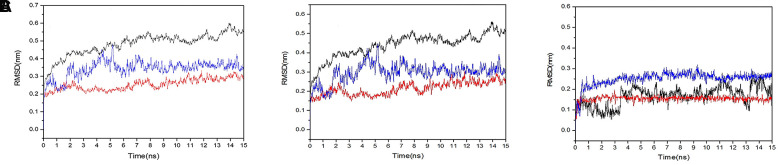
**RMSD plot during molecular dynamics simulations.** (**A**) The RMSD of protein-protein. (**B**) The RMSD of back bond-back bond. (**C**) The RMSD of ligand-ligand (blue polygonal line means licorice glycoside E and S protein, red polygonal line means xambioona and ACE2, black polygonal line means licorice glycoside E and Mpro).

We obtained the protein Calpha root-mean-square deviation (RMSD) data of licorice glycoside E and Spro, xambioona and ACE2, as well as licorice glycoside E and Mpro in terms of proteins, skeletons and ligands. Compared with the first frame in Figure 10A, the composite protein of xambioona and ACE2 became stable after 1 ns, while the stability of licorice glycoside E and Spro after 2 ns was 0.25 and 0.31, respectively, and the final licorice glycoside E and Mpro was stable at 6 ns and the deviation was 0.52. Meanwhile, the backbone of xambioona and ACE2 was also a stable system as shown in Figure 10B, with a deviation of 0.2, while the other two systems tended to be stable after 6 ns. Moreover, from Figure 10C, the system of licorice glycyrrhizin E and S protein reached equilibrium at 0.2 ns with the deviation of 0.23. Xambioona and ACE2 also reached equilibrium at 0.2 ns and maintained up to 50 ns, with the deviation of 0.15. Licorice glycoside E and Mpro reached a balance at 3.5 ns, with a deviation of 0.15. These findings all showed that a stable conformation has been achieved in the process of MD simulation.

## DISCUSSION

COVID-19 is classified in "epidemic disease" in TCM due to its strong infectiousness, rapid transmission and high mortality rate. The main disease location is in the lung with the characteristics of "dampness, poison and epidemic". In TCM theory, the properties of Traditional Chinese drugs are divided into four properties: cool, cold, warm and heat. In addition, there are also some herbs called neutral, whose cold or hot nature is not obvious and their actions are relatively mild. The analysis of anti-COVID-19 traditional Chinese herbal formulas based on data mining showed that the properties of traditional Chinese herbs for COVID-19 are mainly warm, cold and neutral, and mainly belongs to lung, stomach and spleen meridians [17]. Ephedra is mostly featured as warm and Glycyrrhiza featured as neutral in four natures. The Compendium of Materia Medica also mentioned that "Ephedra is a special traditional Chinese herb for the lung meridian". According to the Asian history, both Ephedra and Glycyrrhiza have been used for the treatment of lung diseases for decades.

In our study, TNF, FOS and IL-2 were selected as the key targets in mapping relation analysis between Glycyrrhiza and Ephedra. A retrospective multicentre study of 150 novel coronavirus pneumonia patients suggested that virus-activated "cytokine storm syndrome" might be involved in COVID-19 mortality [50]. Meanwhile, increased serum levels of IL-2, TNF-α, IL-7, granulocyte-colony stimulating factor and interferon-γ inducible protein 10 (CXCL10) are associated with COVID-19 disease severity [3].

Activation of the TNF/TNFR pathway affects the replication of human immunodeficiency virus 1 (HIV-1). Controlling immune activation and disturbing viral replication by controlling the TNF/TNFR pathway is called anti-TNF therapy [51]. IL2 is an essential protein for T cell proliferation and regulation of immune responses [52, 53]. FOS participates in many biological processes such as positive regulation of transcription by the RNAPII promoter and signal transduction. RNAPII is a key protein in virus transcription [54, 55]. In addition, the cAMP signalling pathway was indicated to be the most important signalling pathway in EG pathway enrichment, and EG could also act on the PI3K-Akt, JAK-STAT and chemokine signalling pathways. They are involved in transcription, translation of cytokines and growth factors, immune and cancer related regulations [56–60]. Among them, chemokines could induce the directional chemotaxis of cells (especially white blood cells), which are essential for inflammatory immune responses [60]. Meanwhile, enrichment of EG anti-COVID-19 in the PI3K-Akt signalling pathway made EG related to autophagy. Many positive-strand RNA viruses have been shown to benefit from the autophagy pathway [61]. For example, in dengue virus (DENV) infection, autophagy is utilized in viral entry, translation and replication [62].

The process of SARS-CoV-2 infection could be divided into three steps: attachment and cell entry (S protein and ACE2) [63, 64], replication and transcription (Mpro) [65], the assembly and release of mature **virus**. Baicalin [66], Lopinavir [67] and Arbidol [68] showed good therapeutic effects on SARS-CoV, so they were used as positive control compounds in SARS-CoV-2 infection. In molecular docking and molecular dynamics simulation, we found that 112 active compounds could bind to Mpro, S protein and ACE2 automatically, the binding energies of active compounds were comparable with these positive control compounds. In terms of binding energy, the affinity of licorice glycoside E was better than them with Mpro and S protein; xambioona had better binding affinity with ACE2. The results indicated that the EG-active compounds have good binding activities with Mpro, ACE2 and S proteins. In terms of molecular dynamics, RMSD stability is essential to infer good binding affinity [69]. The complex of Licorice glycoside E and Xambioona with Mpro, S protein and ACE2 showed stable RMSD in the simulated results. So, we identified at least one active compound could inhibit viral infection in the first two steps. In the first stage- attachment and entry, licorice glycyrrhizin E showed steady binding to S and ACE proteins to inhibit host cells infection. In addition, xambioona could bind to the zinc ions area of ACE2 protein, being able to affect the activity of ACE2 and reduce the infection via S protein; In the second stage-replication and transcription, licorice glycyrrhizin E could form a stable combination with Mpro in its activity sites to suppress viral replication and transcription. Licorice glycoside E and xambioona may be the key compounds in the treatment of novel coronavirus pneumonia.

The EG pair has been used to treat other virus infections. The main ingredients Ephedra L-ephedrine and D-pseudo- ephedrine could significantly inhibit the proliferation of influenza A virus, down-regulate the level of TNF- α, significantly attenuate lung injury as well as improve immune function [30]. A randomized trial shows that, maxingshigan-yinqiaosan (composed of 12 Chinese herbal medicines, including honey-fried Ephedra) could be used to H1N1 influenza virus infection. The pairing of Ephedra and Gypsum Fibrosum may have a therapeutic effect on COVID-19 based on the pediatric COVID-19 guidelines [70]. Glycyrrhiza decreased the level of hydroxyproline, reduced pulmonary inflammatory and fibrotic indices *in vivo*, suggesting the prevention and/or treatment of inflammation and pulmonary fibrosis [71]. Glycyrrhiza has been also found to have antiviral activity against SARS-CoV, respiratory syncytial virus and HIV-1 *in vitro* [20, 72, 73]. Glycyrrhizin (active single component from Glycyrrhiza) could significantly inhibit the replication of SARS-CoV, inhibit the virus adsorption and penetration in Vero cells and reduce airway congestion, therefore relieve lung symptoms [22]. Both *in vitro* and *in vivo* studies showed that the combination of Ephedra and Glycyrrhiza could increase anti-inflammatory effects (reducing the levels of TNF- α, IL-1 β and PGE2, regulating Th1/Th2 balance), had no toxicity antagonism, increased efficacy, and increased the contents of several important active compounds including ephedrine, pseudoephedrine, glycyrrhizic acid, glycyrrhizin and so on [74–77]. Both Ephedra and Glycyrrhiza have therapeutic effects on COVID-19, so EG may be more beneficial to COVID-19 treatment, although these findings need to be further verified through experiments.

## CONCLUSIONS

In conclusion, we proposed in our study that EG might be effective against COVID-19 through multiple pathways and targets by network pharmacological analysis, computer molecular docking and molecular dynamics approaches. Licorice glycoside E and xambioona may be the key compounds in the treatment of COVID-19. Therefore, on the basis of our findings, more further experiments to verify the efficacy of EG will accelerate drug development process for COVID-19.

## MATERIALS AND METHODS

### Screening of active ingredients

The Traditional Chinese Medicine Systems Pharmacology Database (TCMSP, http://lsp.nwu.edu.cn/), a unique system pharmacology platform devised for Chinese herbal medicines, was applied to collect the active ingredients of Ephedra-Glycyrrhiza (EG). To make our study more solid and to identify applications for later drug development, we considered two parameters to select active ingredients. One parameter is the Drug-likeness evaluation (DL), which is used to appraise whether a compound is chemically applicable for the drug; the other parameter is the oral bioavailability (OB), which represents the fraction of the oral dose of a bioactive compound that can enter the systemic circulation. We set the OB to more than 30% and the DL to more than 0.18 as the parameters affecting the pharmacodynamics and pharmacokinetic profiles that ultimately affect each compound’s absorption, distribution, metabolism and excretion (ADME) properties. According to the literature, two ADME-related models were inclusive of the evaluation of OB and DL. They were applied to identify the potential bioactive compounds of EG.

### Identification of drug targets and COVID-19 targets

We searched for the drug targets, the channel tropism of the plant, and the natural properties of the EG compounds in the Encyclopedia of Traditional Chinese Medicine (ETCM, http://www.tcmip.cn/ETCM/). It provides comprehensive and standardized information for the commonly used traditional Chinese herbs and formulas of TCM as well as their ingredients. GeneCards was used to combine the relevant literature to generate a pool of COVID-19 targets with the keywords “novel coronavirus pneumonia”. The collected EG targets were placed on the Metascape platform (https://metascape.org/gp/index.html) to obtain the clustered PPI network by the MCODE algorithm, which was presented after modification by Cytoscape.

### Enrichment analysis of drugs (EG)

To investigate the identified EG targets at the functional level, the Gene Ontology (GO) terms and Kyoto Encyclopedia of Genes and Genomes (KEGG) terms were applied to the annotated proteins using p-value <0.05 as the restriction. Additionally, the background species was defined as "Homo sapiens", and the target genes were defined with their official gene symbols. The top 15 GO pathways in gene rich factor as well as the No. 1 pathway (the cAMP signalling pathway) among all of the KEGG pathways were retained to explore the role of the drugs *in vivo*. Then, RStudio and PathVisio software was used to visualize the results analysis.

### Relevant disease analysis of EG

The Genetic Association Database Disease Analysis (GAD Disease Analysis) was performed using the Database for Annotation, Visualization and Integrated Discovery (DAVID, https://david.ncifcrf.gov/) with the limitation of a p-value < 0.05. Cytoscape (http://cytoscape.org/,ver.3.7.2) was used to construct the Target-GAD Disease network.

### Relationship map construction

The target information of EG and COVID-19 were imported into Cytoscape 3.7.2 software (http://www.cytoscape.org/). An online tool, Venny 2.1 (https://bioinfogp.cnb.csic.es/tools/venny/), was used to construct the Drug-Target-Disease relationship map with the thresholds of “Homo sapiens”.

### Enrichment analysis of EG on anti-COVID-19 targets

GO enrichment and pathway enrichment were used to demonstrate how EG compounds may act on COVID-19. To study the specific role of EG in the pathogenesis of the virus *in vivo*, influenza virus was taken as an example. The obtained EG on the anti-COVID-19 targets were mapped into influenza virus replication and transcription, and the specific action pathway of EG involved in influenza virus infection was demonstrated. WikiPathways is a database of biological pathways, where the novel coronavirus pneumonia related pathways have been collected since March 23rd. During SARS-CoV-2 infection, Type I interferons related innate immune response activation was used to map relative targets.

### Compounds-virus network construction

To further explore the mechanism of EG on viral infection-related diseases, the Drugbank database (https://www.drugbank.ca/) was used to obtain the relevant targets of virus-infected diseases with "inflammation" and "virus infection" as keywords, and visualized with the same method in Cytoscape. The proteins closely related to SARS-CoV-2 (ACE2, S protein, Mpro) were selected and amplified to present their interaction with EG targets.

### Molecular docking

The 3D structure of these targets: Mpro (PDB ID:6LU7), S protein (PDB ID:6VSB) and ACE2 (PDB ID:1R42) were downloaded from the protein data bank (PDB) database (https://www.rcsb.org/) [78]. The ligand and water macromolecule in these targets were removed and the hydrogen atoms were added with pymol2.3. The targets were set to rigid and saved as pdbqt file format by AutoDock Tools 1.5.6. In order to gain insight into the potential of EG compounds, based on therapeutic effects on COVID-19, baicalin (CID:64982), lopinavir (CID:92727) and arbidol (CID:131411) were selected in this study as positive control to compare binding energy and molecular docking against COVID-19. A total of 112 active compounds of EG were selected from the TCMSP database (Supplementary Tables 1, 2) and the positive control compounds were downloaded from Pubchem (https://pubchem.ncbi.nlm.nih.gov/). These compounds were energy minimized and saved as mol2 file format using ChemOffice software. Moreover, all compounds were determined as the root, we selected the twisted key, set them to flexible and saved them as pdbqt files through AutoDock Tools 1.5.6. Finally, Molecular docking was performed using Vina. Based on the top one minimal binding energy of each target, xambioona, licorice and glycoside E were selected for further analysis of their binding mode, binding affinity and critical interactions using pymol2.3 and ligplot2.2.

### Molecular dynamics simulation

After docking, the compound with the highest binding energy of each target was simulated by an MD simulation to check the stability of the compound in the binding pocket. Then, the molecular dynamics simulation was performed using the Gromacs 2019.1 software package, the gromos54a7_atb.ff force field and the simple point charge (SPC216) model. To ensure the total charge neutrality of the simulated system, the corresponding amount of sodium ions were added to the three systems to replace water molecules to produce solvent boxes of the appropriate size. Then, the periodic boundary condition (PBC) was applied in all three directions of the system. Using the gromos54a7_atb force field, the force field parameters of the whole atom were obtained from the ATB website (http://atb.uq.edu.au/). Initially, the energy of 50000 steps of the whole system was minimized by (EM) at 300 K. Subsequently, through the MD simulation of position constraints, then through the NVT ensemble (constant number, volume and temperature of particles), and finally through the NPT ensemble (constant number, pressure and temperature of particles), we balanced the enzymes, ligand molecules and solvents.

### Availability of data and materials

The data that support the findings of this study are available from the corresponding author upon reasonable request.

## Supplementary Material

Supplementary Table 1

Supplementary Table 2

Supplementary Table 3

Supplementary Table 4

Supplementary Table 5
