# Supplementary material for: Chemical composition and pharmacological mechanism of ephedra-glycyrrhiza drug pair against coronavirus disease 2019 (COVID-19)
Source: Aging (Albany NY). 2021 Feb 13;13(4):4811–30. doi: 10.18632/aging.202622 (PMC7950231; doi:10.18632/aging.202622)
Supplement: Supplementary Table 1 [file aging-13-202622-s001.pdf]

Supplementary Table 1. Active compounds of *ephedra*.

Mol ID	Molecule Name	OB (%)	DL	HL
**MOL010788**	leucopelargonidin	57.97	0.24	1.38
**MOL002823**	Herbacetin	36.07	0.27	14.8
**MOL010489**	Resivit	30.84	0.27	1.44
**MOL000422**	kaempferol	41.88	0.24	14.74
**MOL004798**	delphinidin	40.63	0.28	1.16
**MOL000098**	quercetin	46.43	0.28	14.4
**MOL000006**	luteolin	36.16	0.25	15.94
**MOL000358**	beta-sitosterol	36.91	0.75	5.36
**MOL000449**	Stigmasterol	43.83	0.76	5.57
**MOL000492**	(+)-catechin	54.83	0.24	0.61
**MOL001494**	Mandenol	42	0.19	5.39
**MOL001506**	Supraene	33.55	0.42	2.72
**MOL001755**	24-Ethylcholest-4-en-3-one	36.08	0.76	5.49
**MOL001771**	poriferast-5-en-3beta-ol	36.91	0.75	5.07
**MOL002881**	Diosmetin	31.14	0.27	16.34
**MOL004328**	naringenin	59.29	0.21	16.98
**MOL004576**	taxifolin	57.84	0.27	14.41
**MOL005043**	campest-5-en-3beta-ol	37.58	0.71	4.43
**MOL005190**	eriodictyol	71.79	0.24	15.81
**MOL005573**	Genkwanin	37.13	0.24	16.1
**MOL005842**	Pectolinarigenin	41.17	0.3	16.56
**MOL007214**	(+)-Leucocyanidin	37.61	0.27	0.9
**MOL011319**	Truflex OBP	43.74	0.24	4.9
