# Supplementary material for: Chemical composition and pharmacological mechanism of ephedra-glycyrrhiza drug pair against coronavirus disease 2019 (COVID-19)
Source: Aging (Albany NY). 2021 Feb 13;13(4):4811–30. doi: 10.18632/aging.202622 (PMC7950231; doi:10.18632/aging.202622)
Supplement: Supplementary Table 2 [file aging-13-202622-s002.docx]

**Supplementary Table 2. Active compounds of *glycyrrhiza.***

| Mol ID | Molecule Name | OB (%) | DL | HL |
| --- | --- | --- | --- | --- |
| MOL001484 | Inermine | 75.18 | 0.54 | 11.72 |
| MOL001792 | DFV | 32.76 | 0.18 | 17.89 |
| MOL000211 | Mairin | 55.38 | 0.78 | 8.87 |
| MOL002311 | Glycyrol | 90.78 | 0.67 | 9.85 |
| MOL000239 | Jaranol | 50.83 | 0.29 | 15.5 |
| MOL002565 | Medicarpin | 49.22 | 0.34 | 8.46 |
| MOL000354 | isorhamnetin | 49.6 | 0.31 | 14.34 |
| MOL000359 | sitosterol | 36.91 | 0.75 | 5.37 |
| MOL003656 | Lupiwighteone | 51.64 | 0.37 | 15.63 |
| MOL003896 | 7-Methoxy-2-methyl isoflavone | 42.56 | 0.2 | 16.89 |
| MOL000392 | formononetin | 69.67 | 0.21 | 17.04 |
| MOL000417 | Calycosin | 47.75 | 0.24 | 17.1 |
| MOL000422 | kaempferol | 41.88 | 0.24 | 14.74 |
| MOL004328 | naringenin | 59.29 | 0.21 | 16.98 |
| MOL004805 | (2S)-2-[4-hydroxy-3-(3-methylbut-2-enyl)phenyl]-8,8-dimethyl-2,3-dihydropyrano[2,3-f]chromen-4-one | 31.79 | 0.72 | 14.82 |
| MOL004806 | euchrenone | 30.29 | 0.57 | 15.89 |
| MOL004808 | glyasperin B | 65.22 | 0.44 | 16.1 |
| MOL004810 | glyasperin F | 75.84 | 0.54 | 15.64 |
| MOL004811 | Glyasperin C | 45.56 | 0.4 | 3.13 |
| MOL004814 | Isotrifoliol | 31.94 | 0.42 | 7.91 |
| MOL004815 | (E)-1-(2,4-dihydroxyphenyl)-3-(2,2-dimethylchromen-6-yl)prop-2-en-1-one | 39.62 | 0.35 | 16.16 |
| MOL004820 | kanzonols W | 50.48 | 0.52 | 0.15 |
| MOL004824 | (2S)-6-(2,4-dihydroxyphenyl)-2-(2-hydroxypropan-2-yl)-4-methoxy-2,3-dihydrofuro[3,2-g]chromen-7-one | 60.25 | 0.63 | 4.31 |
| MOL004827 | Semilicoisoflavone B | 48.78 | 0.55 | 17.02 |
| MOL004828 | Glepidotin A | 44.72 | 0.35 | 16.09 |
| MOL004829 | Glepidotin B | 64.46 | 0.34 | 15.98 |
| MOL004833 | Phaseolinisoflavan | 32.01 | 0.45 | 2.66 |
| MOL004835 | Glypallichalcone | 61.6 | 0.19 | 17.01 |
| MOL004838 | 8-(6-hydroxy-2-benzofuranyl)-2,2-dimethyl-5-chromenol | 58.44 | 0.38 | 8.71 |
| MOL004841 | Licochalcone B | 76.76 | 0.19 | 17.02 |
| MOL004848 | licochalcone G | 49.25 | 0.32 | 15.75 |
| MOL004849 | 3-(2,4-dihydroxyphenyl)-8-(1,1-dimethylprop-2-enyl)-7-hydroxy-5-methoxy-coumarin | 59.62 | 0.43 | 0.69 |
| MOL004855 | Licoricone | 63.58 | 0.47 | 16.37 |
| MOL004856 | Gancaonin A | 51.08 | 0.4 | 16.82 |
| MOL004857 | Gancaonin B | 48.79 | 0.45 | 16.49 |
| MOL004860 | licorice glycoside E | 32.89 | 0.27 | 25.39 |
| MOL004863 | 3-(3,4-dihydroxyphenyl)-5,7-dihydroxy-8-(3-methylbut-2-enyl)chromone | 66.37 | 0.41 | 15.81 |
| MOL004864 | 5,7-dihydroxy-3-(4-methoxyphenyl)-8-(3-methylbut-2-enyl)chromone | 30.49 | 0.41 | 14.99 |
| MOL004866 | 2-(3,4-dihydroxyphenyl)-5,7-dihydroxy-6-(3-methylbut-2-enyl)chromone | 44.15 | 0.41 | 16.77 |
| MOL004879 | Glycyrin | 52.61 | 0.47 | 1.31 |
| MOL004882 | Licocoumarone | 33.21 | 0.36 | 9.66 |
| MOL004883 | Licoisoflavone | 41.61 | 0.42 | 16.09 |
| MOL004884 | Licoisoflavone B | 38.93 | 0.55 | 15.73 |
| MOL004885 | licoisoflavanone | 52.47 | 0.54 | 15.67 |
| MOL004891 | shinpterocarpin | 80.3 | 0.73 | 6.5 |
| MOL004898 | (E)-3-[3,4-dihydroxy-5-(3-methylbut-2-enyl)phenyl]-1-(2,4-dihydroxyphenyl)prop-2-en-1-one | 46.27 | 0.31 | 15.24 |
| MOL004903 | liquiritin | 65.69 | 0.74 | 17.96 |
| MOL004904 | licopyranocoumarin | 80.36 | 0.65 | 0.08 |
| MOL004905 | 3,22-Dihydroxy-11-oxo-delta(12)-oleanene-27-alpha-methoxycarbonyl-29-oic acid | 34.32 | 0.55 | 3.56 |
| MOL004907 | Glyzaglabrin | 61.07 | 0.35 | 21.2 |
| MOL004908 | Glabridin | 53.25 | 0.47 | 0.03 |
| MOL004910 | Glabranin | 52.9 | 0.31 | 16.24 |
| MOL004911 | Glabrene | 46.27 | 0.44 | 3.63 |
| MOL004912 | Glabrone | 52.51 | 0.5 | 16.09 |
| MOL004913 | 1,3-dihydroxy-9-methoxy-6-benzofurano[3,2-c]chromenone | 48.14 | 0.43 | 8.87 |
| MOL004914 | 1,3-dihydroxy-8,9-dimethoxy-6-benzofurano[3,2-c]chromenone | 62.9 | 0.53 | 9.32 |
| MOL004915 | Eurycarpin A | 43.28 | 0.37 | 17.1 |
| MOL004917 | glycyroside | 37.25 | 0.79 | 14.62 |
| MOL004924 | (-)-Medicocarpin | 40.99 | 0.95 | 13.2 |
| MOL004935 | Sigmoidin-B | 34.88 | 0.41 | 14.49 |
| MOL004941 | (2R)-7-hydroxy-2-(4-hydroxyphenyl)chroman-4-one | 71.12 | 0.18 | 18.09 |
| MOL004945 | (2S)-7-hydroxy-2-(4-hydroxyphenyl)-8-(3-methylbut-2-enyl)chroman-4-one | 36.57 | 0.32 | 17.95 |
| MOL004948 | Isoglycyrol | 44.7 | 0.84 | 6.69 |
| MOL004949 | Isolicoflavonol | 45.17 | 0.42 | 15.55 |
| MOL004957 | HMO | 38.37 | 0.21 | 16.56 |
| MOL004959 | 1-Methoxyphaseollidin | 69.98 | 0.64 | 9.53 |
| MOL004961 | Quercetin der. | 46.45 | 0.33 | 16.61 |
| MOL004966 | 3'-Hydroxy-4'-O-Methylglabridin | 43.71 | 0.57 | -0.61 |
| MOL000497 | licochalcone a | 40.79 | 0.29 | 16.2 |
| MOL004974 | 3'-Methoxyglabridin | 46.16 | 0.57 | 0.52 |
| MOL004978 | 2-[(3R)-8,8-dimethyl-3,4-dihydro-2H-pyrano[6,5-f]chromen-3-yl]-5-methoxyphenol | 36.21 | 0.52 | -0.13 |
| MOL004980 | Inflacoumarin A | 39.71 | 0.33 | 2.31 |
| MOL004985 | icos-5-enoic acid | 30.7 | 0.2 | 5.28 |
| MOL004988 | Kanzonol F | 32.47 | 0.89 | 9.98 |
| MOL004989 | 6-prenylated eriodictyol | 39.22 | 0.41 | 16.52 |
| MOL004990 | 7,2',4'-trihydroxy－5-methoxy-3－arylcoumarin | 83.71 | 0.27 | 0.99 |
| MOL004991 | 7-Acetoxy-2-methylisoflavone | 38.92 | 0.26 | 17.49 |
| MOL004993 | 8-prenylated eriodictyol | 53.79 | 0.4 | 15.7 |
| MOL004996 | gadelaidic acid | 30.7 | 0.2 | 5.25 |
| MOL000500 | Vestitol | 74.66 | 0.21 | 3 |
| MOL005000 | Gancaonin G | 60.44 | 0.39 | 16.13 |
| MOL005001 | Gancaonin H | 50.1 | 0.78 | 16.64 |
| MOL005003 | Licoagrocarpin | 58.81 | 0.58 | 9.45 |
| MOL005007 | Glyasperins M | 72.67 | 0.59 | 15.57 |
| MOL005008 | Glycyrrhiza flavonol A | 41.28 | 0.6 | 13.71 |
| MOL005012 | Licoagroisoflavone | 57.28 | 0.49 | 19.64 |
| MOL005013 | 18α-hydroxyglycyrrhetic acid | 41.16 | 0.71 | 4.96 |
| MOL005016 | Odoratin | 49.95 | 0.3 | 16.35 |
| MOL005017 | Phaseol | 78.77 | 0.58 | 9.64 |
| MOL005018 | Xambioona | 54.85 | 0.87 | 14.5 |
| MOL005020 | dehydroglyasperins C | 53.82 | 0.37 | 2.75 |
| MOL000098 | quercetin | 46.43 | 0.28 | 14.4 |
