# Supplementary material for: Chemical composition and pharmacological mechanism of ephedra-glycyrrhiza drug pair against coronavirus disease 2019 (COVID-19)
Source: Aging (Albany NY). 2021 Feb 13;13(4):4811–30. doi: 10.18632/aging.202622 (PMC7950231; doi:10.18632/aging.202622)
Supplement: Supplementary Table 3 [file aging-13-202622-s003.pdf]

Supplementary Table 3. Data analysis summary for 25 mapping targets.

Name	Degree	BetweennessCentrality	ClosenessCentrality	ClusteringCoefficient
ALB	39	0.158702	0.73494	0.396761
TNF-a	38	0.083162	0.701149	0.452347
IL-6	37	0.085899	0.709302	0.460961
ERK2	32	0.075523	0.642105	0.522177
CASP3	31	0.041898	0.635417	0.591398
PTGS2	31	0.020453	0.642105	0.608602
CXCL8	30	0.02034	0.628866	0.606897
RELA	27	0.015651	0.616162	0.618234
CREB1	27	0.040129	0.61	0.555556
CCL2	27	0.01732	0.61	0.669516
SIRT1	26	0.03565	0.622449	0.587692
eNOS	25	0.030048	0.61	0.673333
IFN-a	24	0.009279	0.586538	0.688406
GPT	23	0.032022	0.598039	0.533597
HMOX1	22	0.002617	0.580952	0.813853
