# Supplementary material for: Chemical composition and pharmacological mechanism of ephedra-glycyrrhiza drug pair against coronavirus disease 2019 (COVID-19)
Source: Aging (Albany NY). 2021 Feb 13;13(4):4811–30. doi: 10.18632/aging.202622 (PMC7950231; doi:10.18632/aging.202622)
Supplement: Supplementary Table 4 [file aging-13-202622-s004.docx]

**Supplementary Table 4. The binding energy of 112 active compounds binding and protein targets.**

| compounds | relative molecular mass | structure | Molecular Docking Binding Energy (kj/mol) | | |
| --- | --- | --- | --- | --- | --- |
|  |  |  | Mpro | ACE2 | Spike |
| luteolin | 286.25 | 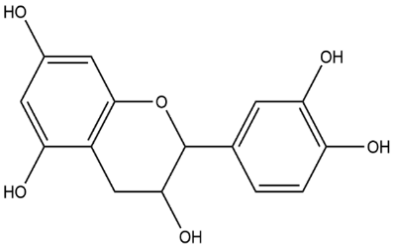 | -30.8096 | -29.8468 | -29.8468 |
| quercetin | 302.25 | 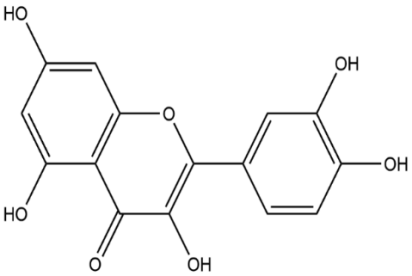 | -20.7002 | -36.1050 | -27.9212 |
| Mairin | 456.78 | 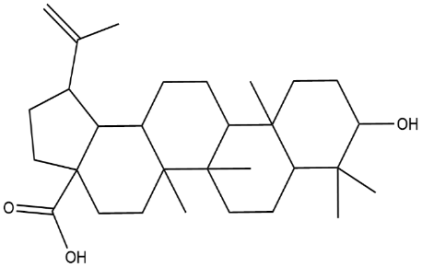 | -31.2910 | -35.6236 | -31.7724 |
| Jaranol | 314.31 | 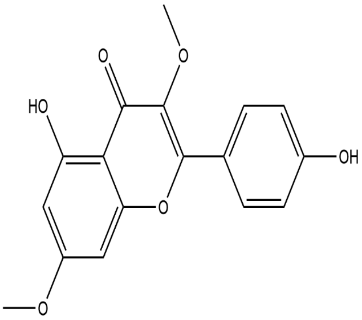 | -32.2538 | -32.2538 | -25.5142 |
| isorhamnetin | 316.28 | 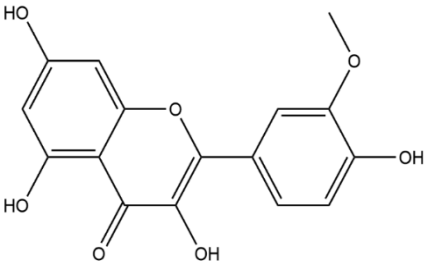 | -31.2910 | -29.3654 | -29.8468 |
| beta-sitosterol | 414.79 | 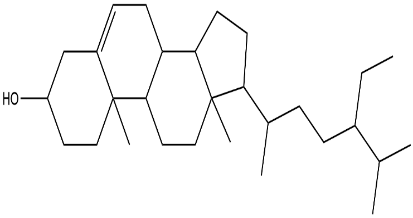 | -30.8096 | -29.3654 | -28.4026 |
| sitosterol | 414.79 | 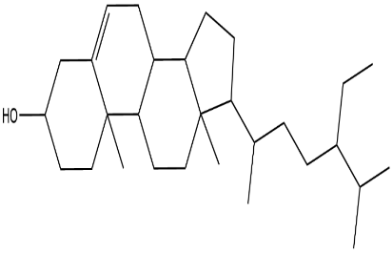 | -30.3282 | -31.7724 | -26.477 |
| formononetin | 268.28 | 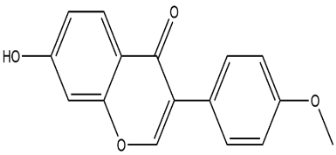 | -31.7724 | -35.6236 | -26.477 |
| Calycosin | 284.28 | 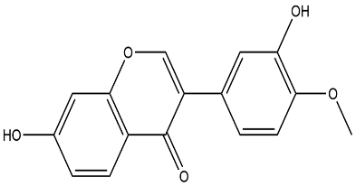 | -31.7724 | -32.7352 | -27.4398 |
| kaempferol | 286.25 | 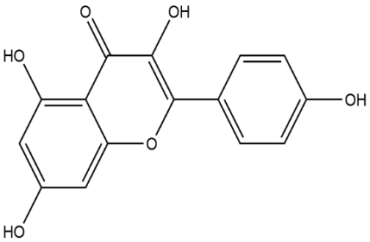 | -31.7724 | -31.7724 | -30.8096 |
| Stigmasterol | 412.77 | 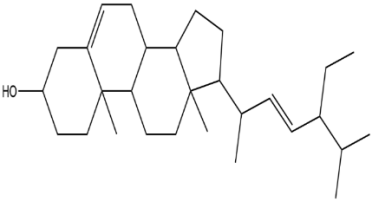 | -30.3282 | -33.698 | -28.884 |
| (+)-catechin | 290.29 | 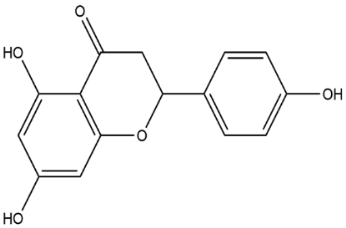 | -23.5886 | -34.1794 | -29.8468 |
| licochalcone a | 338.43 | 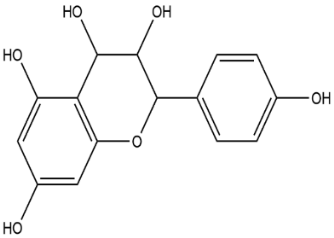 | -29.3654 | -33.698 | -28.4026 |
| Vestitol | 272.32 | 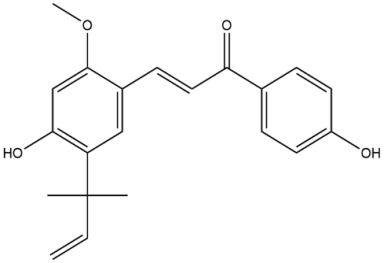 | -31.7724 | -34.1794 | -29.3654 |
| Inermine | 284.28 | 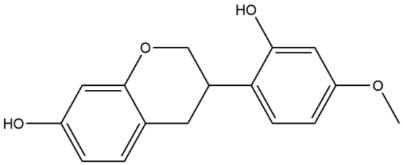 | -29.8468 | -34.1794 | -29.3654 |
| Mandenol | 308.56 | 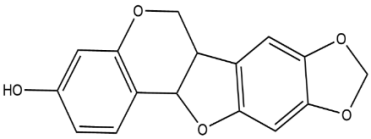 | -32.7352 | -33.2166 | -29.3654 |
| Supraene | 410.8 | 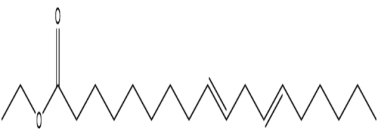 | -32.2538 | -33.2166 | -21.1816 |
| 24-Ethylcholest-4-en-3-one | 412.77 | 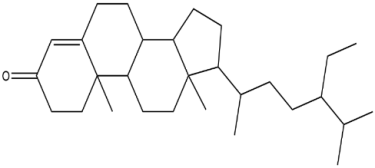 | -30.3282 | -36.5864 | -22.1444 |
| poriferast-5-en-3beta-ol | 414.79 | 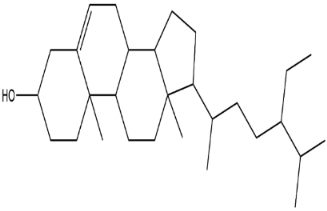 | -20.7002 | -32.7352 | -28.4026 |
| DFV | 256.27 | 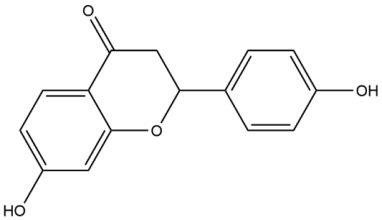 | -30.8096 | -34.6608 | -28.4026 |
| Glycyrol | 366.39 | 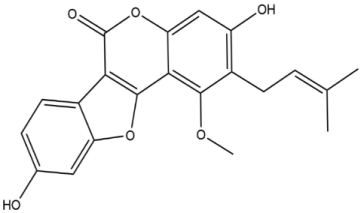 | -29.8468 | -31.7724 | -29.8468 |
| Medicarpin | 270.3 | 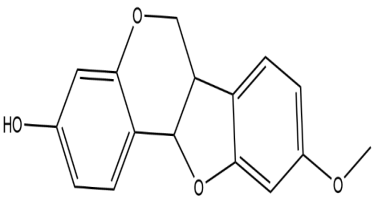 | -32.2538 | -34.1794 | -27.9212 |
| Herbacetin | 302.25 | 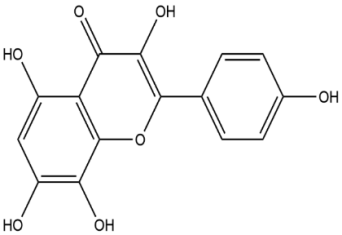 | -33.2166 | -31.2910 | -27.9212 |
| Diosmetin | 300.28 | 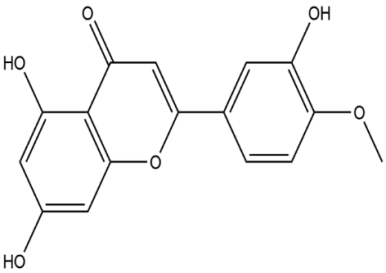 | -29.3654 | -43.8074 | -29.3654 |
| Lupiwighteone | 338.38 | 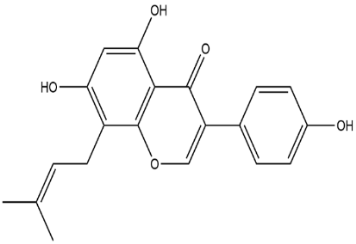 | -31.2910 | -35.1422 | -29.3654 |
| 7-Methoxy-2-methyl isoflavone | 266.31 | 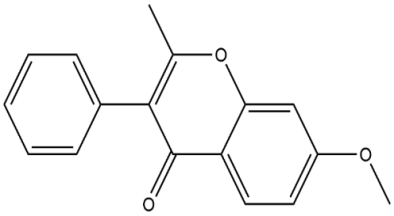 | -28.884 | -33.2166 | -28.884 |
| naringenin | 272.27 | 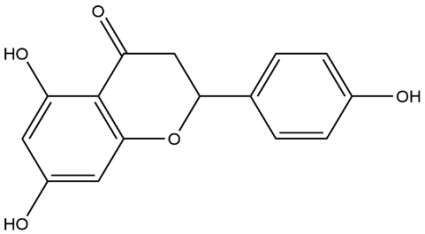 | -29.8468 | -38.512 | -28.884 |
| taxifolin | 304.27 | 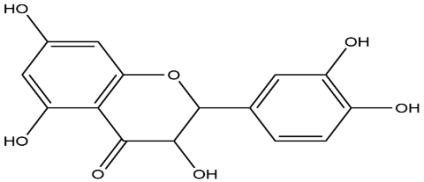 | -29.8468 | -33.698 | -27.9212 |
| delphinidin | 303.26 | 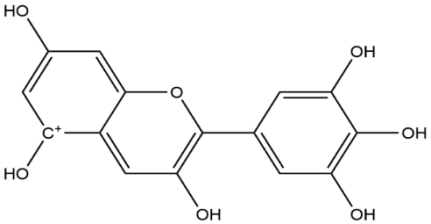 | -30.3282 | -29.8468 | -28.884 |
| euchrenone | 406.56 | 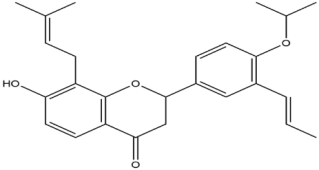 | -28.8840 | -32.2538 | -34.6608 |
| glyasperin B | 370.43 | 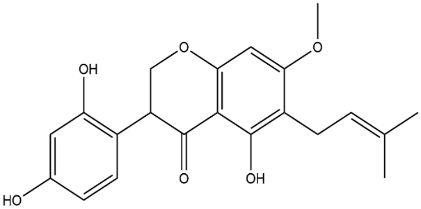 | -32.2538 | -33.6980 | -27.9212 |
| glyasperin F | 354.38 | 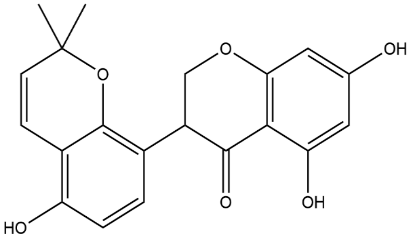 | -29.3654 | -32.7352 | -27.9212 |
| Glyasperin C | 356.45 | 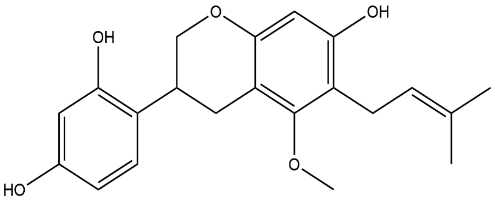 | -33.2166 | -35.6236 | -30.8096 |
| Isotrifoliol | 298.26 | 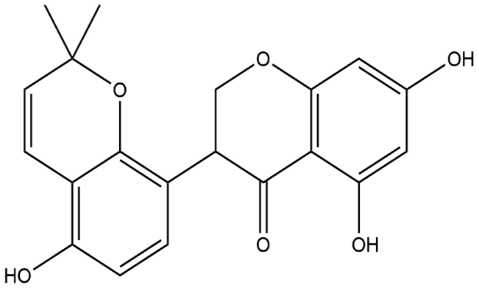 | -30.3282 | -34.1794 | -29.8468 |
| (E)-1-(2,4-dihydroxyphenyl)-3-(2,2-dimethylchromen-6-yl)prop-2-en-1-one | 322.38 | 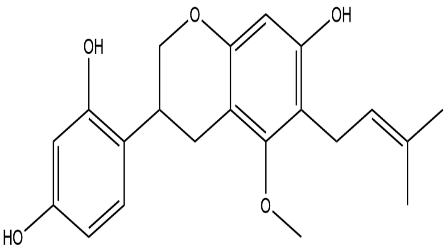 | -30.3282 | -34.6608 | -27.9212 |
| kanzonols W | 336.36 | 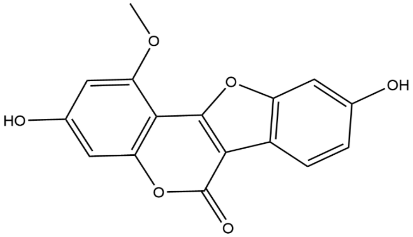 | -29.8468 | -32.2538 | -32.2538 |
| (2S)-6-(2,4-dihydroxyphenyl)-2-(2-hydroxypropan-2-yl)-4-methoxy-2,3-dihydrofuro[3,2-g]chromen-7-one | 384.41 | 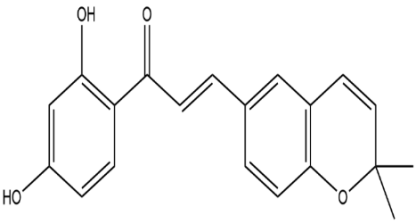 | -29.3654 | -36.1050 | -32.2538 |
| Semilicoisoflavone B | 352.36 | 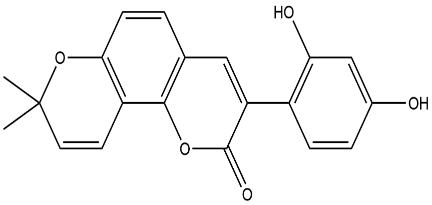 | -34.6608 | -36.1050 | -28.884 |
| Glepidotin A | 338.38 | 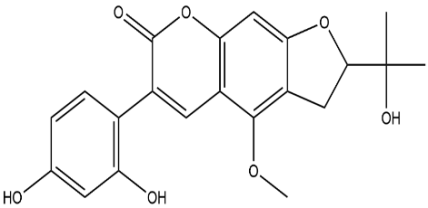 | -33.2166 | -35.1422 | -29.8468 |
| Glepidotin B | 340.4 | 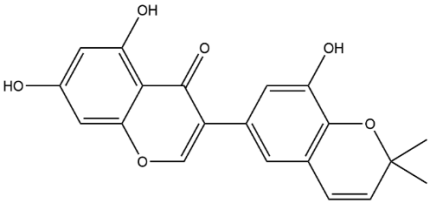 | -31.7724 | -31.7724 | -28.4026 |
| Phaseolinisoflavan | 324.4 | 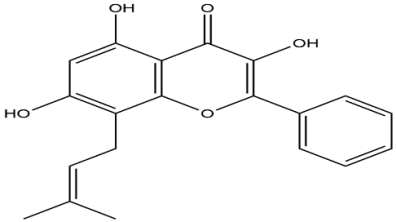 | -31.7724 | -33.2166 | -27.4398 |
| Glypallichalcone | 324.33 | 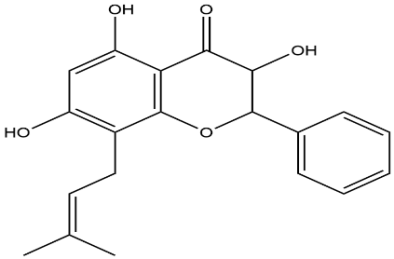 | -29.8468 | -36.105 | -31.291 |
| Licochalcone B | 286.3 | 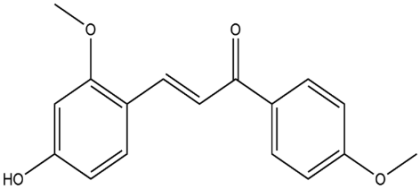 | -34.1794 | -33.698 | -30.8096 |
| licochalcone G | 354.43 | 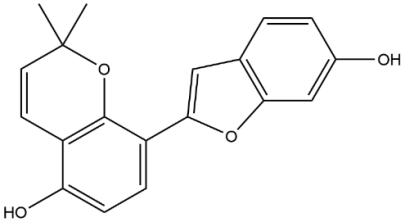 | -35.1422 | -31.291 | -29.3654 |
| 3-(2,4-dihydroxyphenyl)-8-(1,1-dimethylprop-2-enyl)-7-hydroxy-5-methoxy-coumarin | 368.41 | 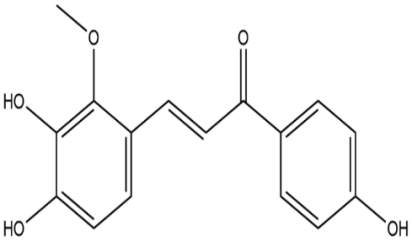 | -36.5864 | -32.2538 | -28.884 |
| Licoricone | 382.44 | 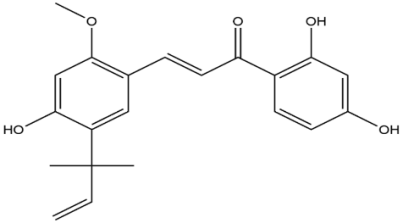 | -34.6608 | -30.8096 | -29.3654 |
| Gancaonin A | 352.41 | 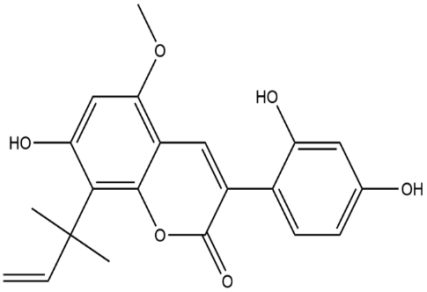 | -33.6980 | -32.7352 | -27.9212 |
| Gancaonin B | 368.41 | 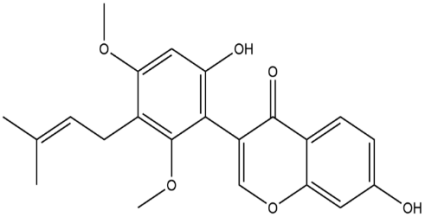 | -31.7724 | -36.1050 | -26.9584 |
| licorice glycoside E | 693.71 | 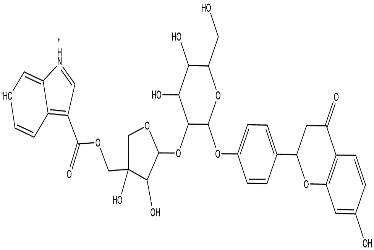 | -40.6500 | -36.1050 | -27.4398 |
| 3-(3,4-dihydroxyphenyl)-5,7-dihydroxy-8-(3-methylbut-2-enyl)chromone | 354.38 | 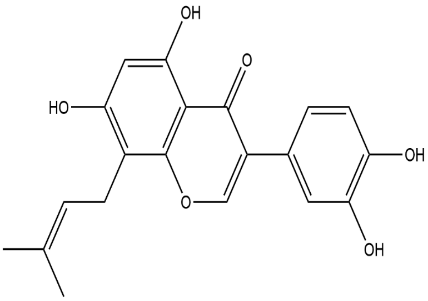 | -30.8096 | -32.7352 | -36.1050 |
| 5,7-dihydroxy-3-(4-methoxyphenyl)-8-(3-methylbut-2-enyl)chromone | 352.41 | 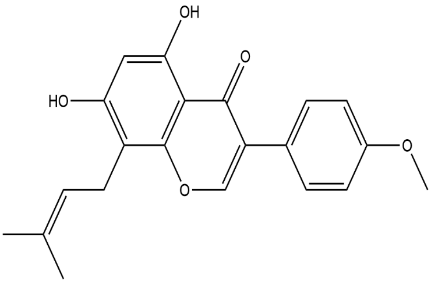 | -32.2538 | -34.6608 | -30.3282 |
| 2-(3,4-dihydroxyphenyl)-5,7-dihydroxy-6-(3-methylbut-2-enyl)chromone | 354.38 | 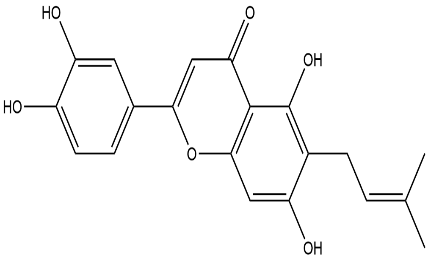 | -30.3282 | -29.8468 | -27.9212 |
| Glycyrin | 382.44 | 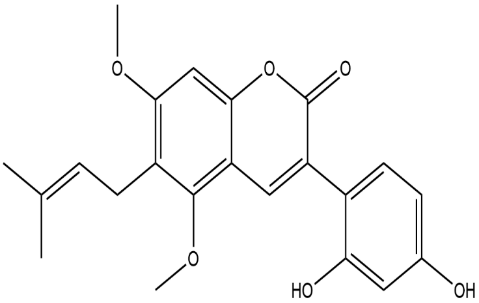 | -32.2538 | -29.8468 | -29.3654 |
| Licocoumarone | 340.4 | 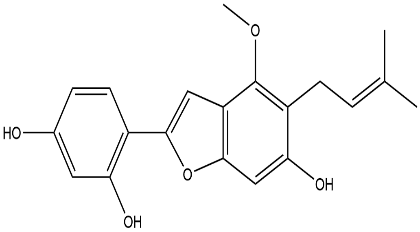 | -31.2910 | -32.7352 | -28.4026 |
| Licoisoflavone | 354.38 | 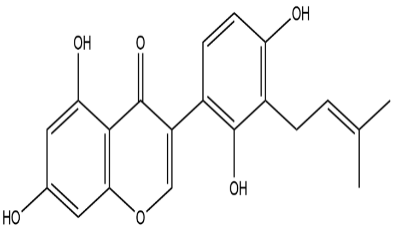 | -30.8096 | -34.6608 | -30.3282 |
| Licoisoflavone B | 352.36 | 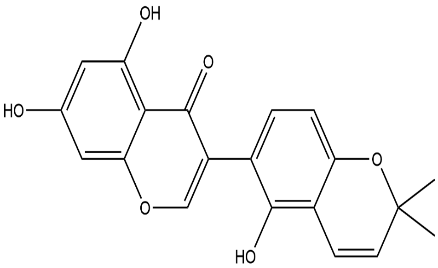 | -30.3282 | -35.1422 | -29.3654 |
| licoisoflavanone | 354.38 | 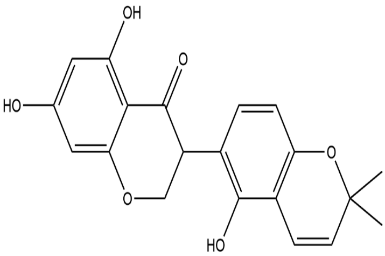 | -31.7724 | -38.0306 | -30.8096 |
| shinpterocarpin | 322.38 | 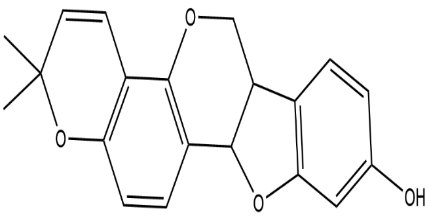 | -39.4748 | -34.6608 | -32.2538 |
| (E)-3-[3,4-dihydroxy-5-(3-methylbut-2-enyl)phenyl]-1-(2,4-dihydroxyphenyl)prop-2-en-1-one | 340.4 | 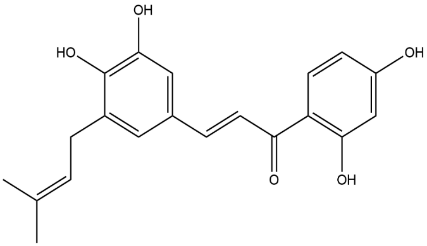 | -32.2538 | -30.8096 | -31.291 |
| liquiritin | 418.43 | 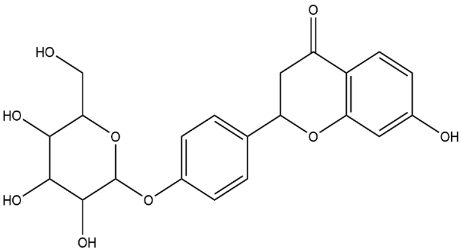 | -31.2910 | -32.2538 | -31.291 |
| licopyranocoumarin | 384.41 | 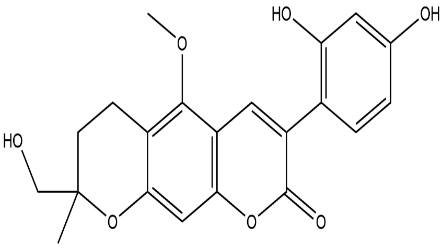 | -33.698 | -32.7352 | -29.3654 |
| 3,22-Dihydroxy-11-oxo-delta(12)-oleanene-27-alpha-methoxycarbonyl-29-oic acid | 512.75 | 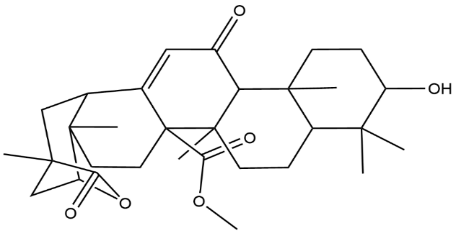 | -30.8096 | -34.6608 | -28.4026 |
| Glyzaglabrin | 298.26 | 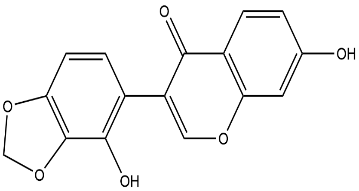 | -29.8468 | -32.7352 | -33.2166 |
| Glabridin | 324.4 | 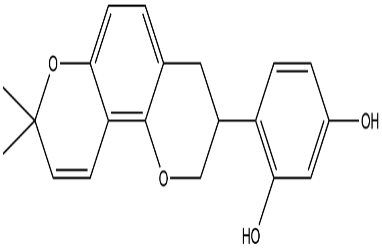 | -31.7724 | -36.105 | -29.3654 |
| Glabranin | 324.4 | 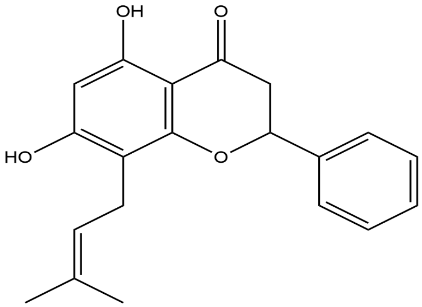 | -33.698 | -36.5864 | -33.698 |
| Glabrene | 322.38 | 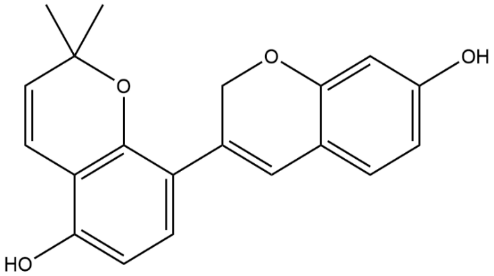 | -34.6608 | -35.6236 | -27.9212 |
| Glabrone | 336.36 | 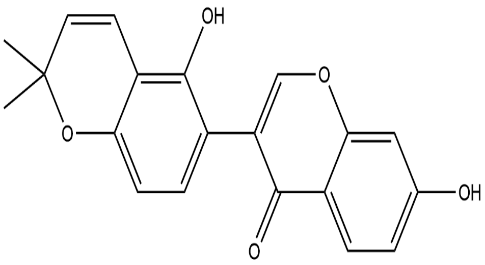 | -35.6236 | -34.6608 | -33.698 |
| 1,3-dihydroxy-9-methoxy-6-benzofurano[3,2-c]chromenone | 298.26 | 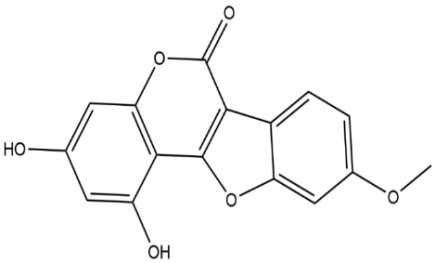 | -32.7352 | -33.2166 | -29.8468 |
| 1,3-dihydroxy-8,9-dimethoxy-6-benzofurano[3,2-c]chromenone | 328.29 | 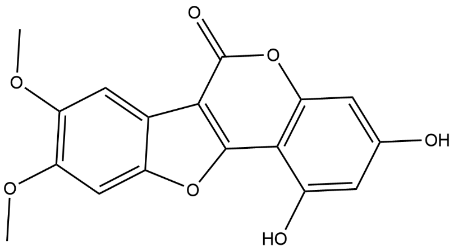 | -35.1422 | -33.698 | -27.4398 |
| Eurycarpin A | 338.38 | 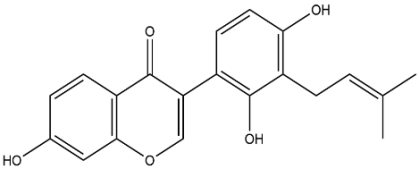 | -34.6608 | -33.698 | -28.4026 |
| glycyroside | 562.57 | 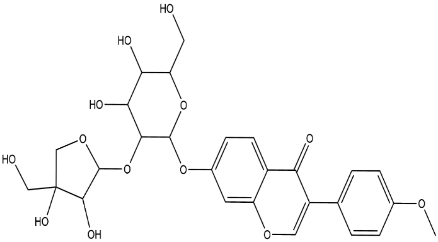 | -34.6608 | -33.2166 | -28.884 |
| (-)-Medicocarpin | 432.46 | 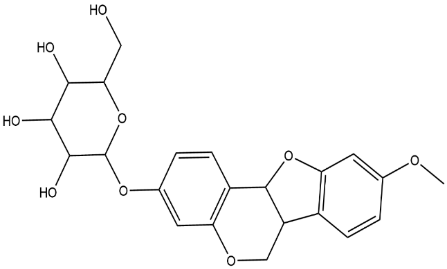 | -31.2910 | -37.0678 | -30.3282 |
| Sigmoidin-B | 356.4 | 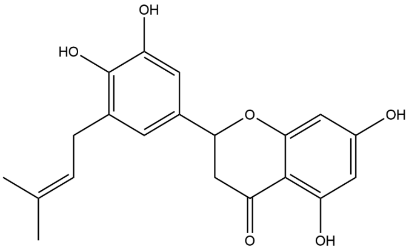 | -33.6980 | -33.6980 | -30.3282 |
| (2R)-7-hydroxy-2-(4-hydroxyphenyl)chroman-4-one | 256.27 | 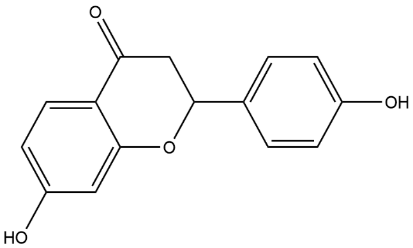 | -31.7724 | -32.7352 | -31.2910 |
| (2S)-7-hydroxy-2-(4-hydroxyphenyl)-8-(3-methylbut-2-enyl)chroman-4-one | 324.4 | 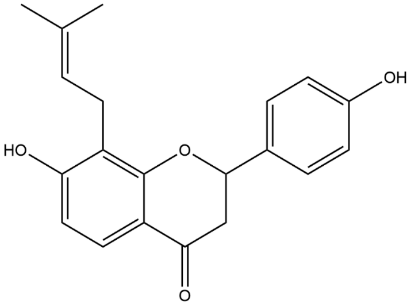 | -32.7352 | -36.1050 | -27.4398 |
| Isoglycyrol | 366.39 | 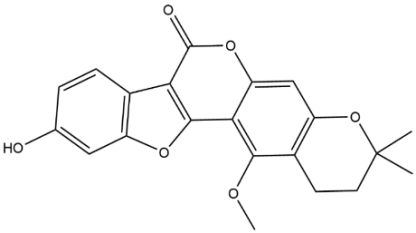 | -34.6608 | -37.5492 | -29.8468 |
| Isolicoflavonol | 354.38 | 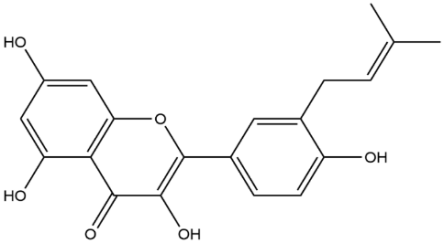 | -32.2538 | -32.7352 | -28.884 |
| HMO | 268.28 | 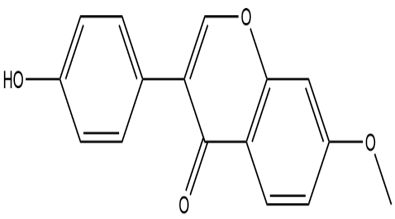 | -30.3282 | -34.6608 | -31.2910 |
| 1-Methoxyphaseollidin | 354.43 | 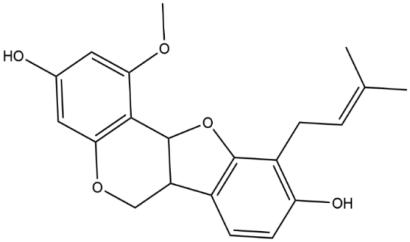 | -31.7724 | -35.1422 | -26.9584 |
| Quercetin der. | 330.31 | 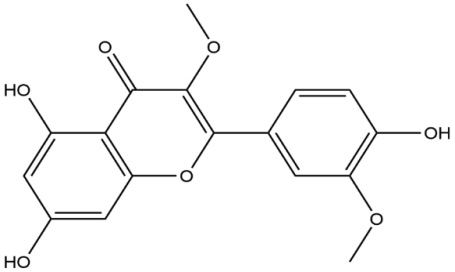 | -37.0678 | -33.6980 | -29.3654 |
| 3'-Hydroxy-4'-O-Methylglabridin | 354.43 | 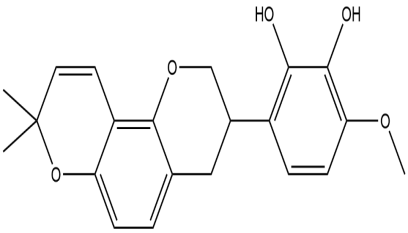 | -35.1422 | -32.7352 | -27.9212 |
| 3'-Methoxyglabridin | 354.43 | 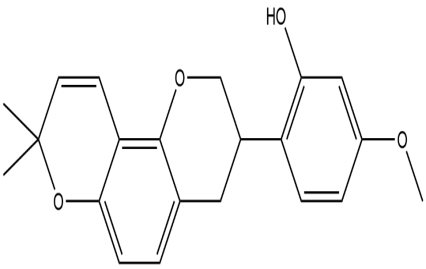 | -34.6608 | -33.6980 | -29.3654 |
| Inflacoumarin A | 322.38 | 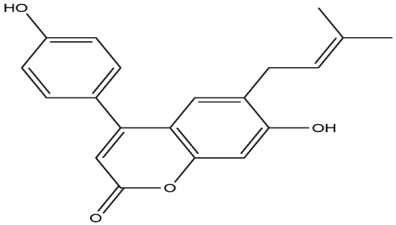 | -35.1422 | -35.6236 | -31.7724 |
| icos-5-enoic acid | 310.58 | 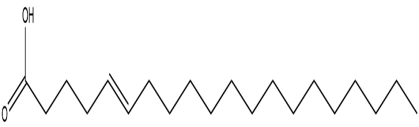 | -34.6608 | -32.7352 | -31.2910 |
| Kanzonol F | 420.54 | 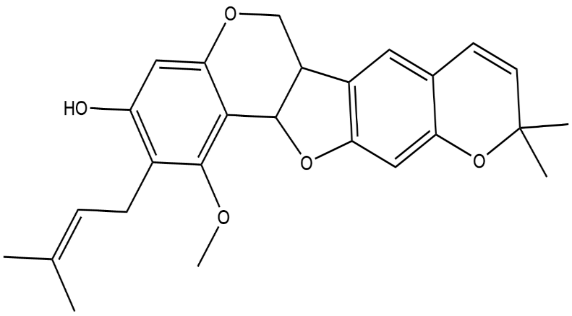 | -35.1422 | -34.6608 | -18.2932 |
| 6-prenylated eriodictyol | 356.4 | 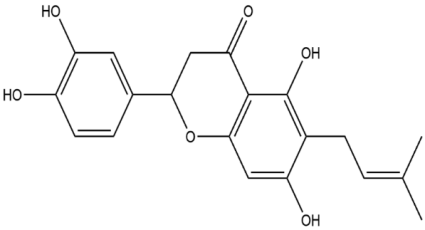 | -28.4026 | -33.6980 | -31.7724 |
| 7,2',4'-trihydroxy－5-methoxy-3－arylcoumarin | 300.28 | 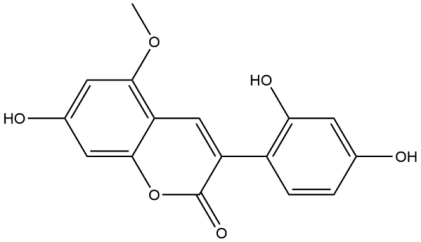 | -33.2166 | -32.2538 | -30.3282 |
| 7-Acetoxy-2-methylisoflavone | 294.32 | 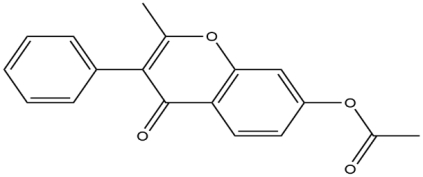 | -31.7724 | -36.5864 | -32.2538 |
| 8-prenylated eriodictyol | 356.4 | 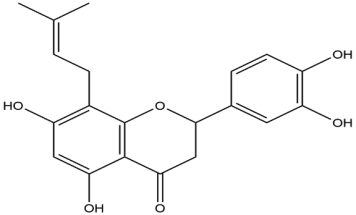 | -37.0678 | -32.7352 | -28.4026 |
| gadelaidic acid | 310.58 | 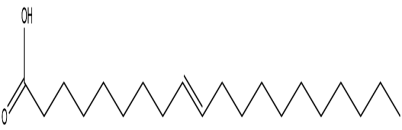 | -34.1794 | -30.8096 | -29.8468 |
| Gancaonin G | 352.41 | 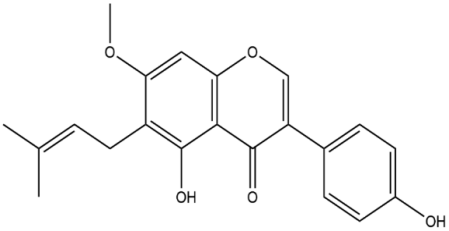 | -34.1794 | -33.2166 | -19.7374 |
| Gancaonin H | 420.49 | 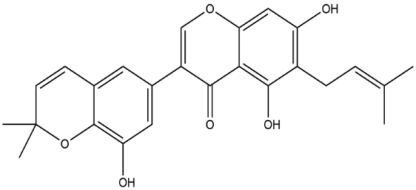 | -34.6608 | -35.6236 | -27.9212 |
| Licoagrocarpin | 338.43 | 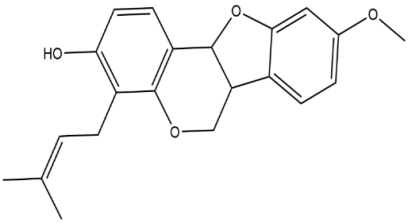 | -19.7374 | -33.6980 | -31.2910 |
| Glyasperins M | 368.41 | 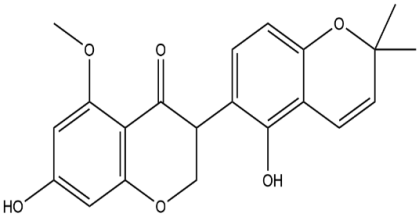 | -34.1794 | -37.0678 | -29.8468 |
| Glycyrrhiza flavonol A | 370.38 | 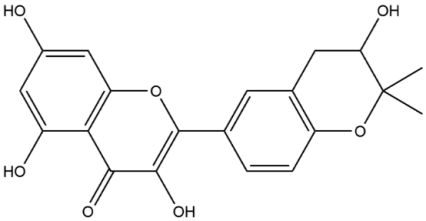 | -33.2166 | -32.2538 | -30.3282 |
| Licoagroisoflavone | 336.36 | 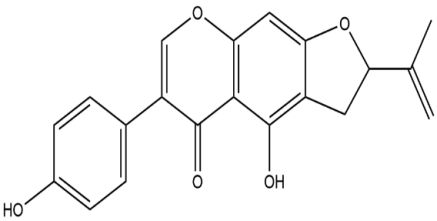 | -31.7724 | -39.9562 | -32.7352 |
| 18α-hydroxyglycyrrhetic acid | 486.76 | 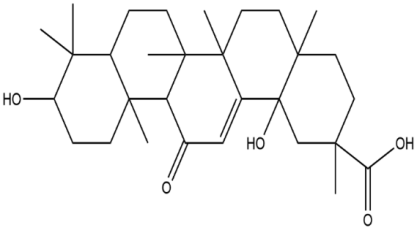 | -32.2538 | -32.2538 | -29.8468 |
| Odoratin | 314.31 | 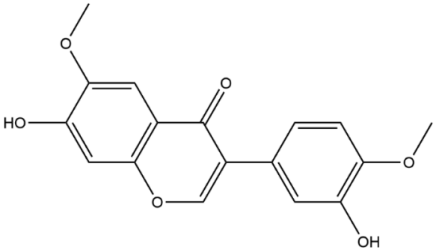 | -34.6608 | -38.0306 | -29.8468 |
| Phaseol | 336.36 | 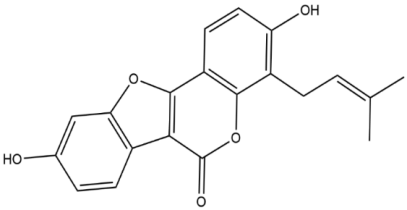 | -23.5886 | -39.4748 | -26.9584 |
| Xambioona | 388.49 | 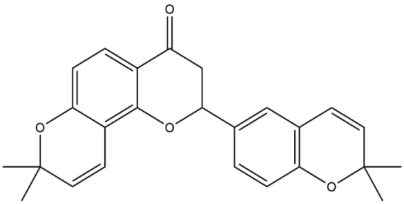 | -29.8468 | -50.5470 | -31.2910 |
| dehydroglyasperins C | 340.4 |  | -38.0306 | -32.2538 | -34.1794 |
| campest-5-en-3beta-ol | 400.76 |  | -31.7724 | -31.7724 | -30.3282 |
| eriodictyol | 288.27 |  | -34.1794 | -32.2538 | -30.8096 |
| Genkwanin | 284.28 |  | -34.1794 | -31.7724 | -29.3654 |
| Pectolinarigenin | 314.31 |  | -31.7724 | -35.6236 | -27.9212 |
| (+)-Leucocyanidin | 306.29 |  | -34.6608 | -35.6236 | -27.4398 |
| Resivit | 306.29 |  | -30.3282 | -34.6608 | -29.3654 |
| Leucopelargonidin | 290.29 |  | -34.6608 | -50.5470 | -28.884 |
| Truflex OBP | 334.5 |  | -36.5864 | -30.3282 | -29.8468 |
