# Supplementary material for: Chemical composition and pharmacological mechanism of ephedra-glycyrrhiza drug pair against coronavirus disease 2019 (COVID-19)
Source: Aging (Albany NY). 2021 Feb 13;13(4):4811–30. doi: 10.18632/aging.202622 (PMC7950231; doi:10.18632/aging.202622)
Supplement: Supplementary Table 5 [file aging-13-202622-s005.pdf]

Supplementary Table 5. The docking results of top one results of each targets and standard drug *Baicalin*, lopinavir and arbidol.

NO	compounds	reference	Docking (binding energy) (kJ/mol)		
			Mpro	ACE2	Spro
M1	*Licorice glycoside E*	[1]	-39.47	-31.15	-36.10
M2	*Xambioona*	[2]	-36.58	-50.50	-34.17
M3	*Baicalin*	[3, 4]	-34.66	-47.25	-31.29
M4	lopinavir	[5]	-38.99	-45.25	-29.84
M5	arbidol	[5]	-26.47	-31.29	-22.14

Supplementary References

1. Nazari S, Rameshrad M, Hosseinzadeh H. Toxicological effects of glycyrrhiza glabra (licorice): a review. Phytother Res. 2017; 31:1635–50. https://doi.org/10.1002/ptr.5893 PMID:28833680

2. Wu C, Liu Y, Yang Y, Zhang P, Zhong W, Wang Y, Wang Q, Xu Y, Li M, Li X, Zheng M, Chen L, Li H. Analysis of therapeutic targets for SARS-CoV-2 and discovery of potential drugs by computational methods. Acta Pharm Sin B. 2020; 10:766–88. https://doi.org/10.1016/j.apsb.2020.02.008 PMID:32292689

3. Chen F, Chan KH, Jiang Y, Kao RY, Lu HT, Fan KW, Cheng VC, Tsui WH, Hung IF, Lee TS, Guan Y, Peiris JS, Yuen KY. *In vitro* susceptibility of 10 clinical isolates of SARS coronavirus to selected antiviral compounds. J Clin Virol. 2004; 31:69–75. https://doi.org/10.1016/j.jcv.2004.03.003 PMID:15288617

4. Deng YF, Aluko RE, Jin Q, Zhang Y, Yuan LJ. Inhibitory activities of baicalin against renin and angiotensin-converting enzyme. Pharm Biol. 2012; 50:401–06. https://doi.org/10.3109/13880209.2011.608076 PMID:22136493

5. Chen J, Ling Y, Xi X, Liu P, Li F, Li T, Shang Z, Wang M, Shen Y, Lu H. Efficacies of lopinavir/ritonavir and abidol in the treatment of novel coronavirus pneumonia. Chinese Journal of Infectious Diseases. 2020; 38:E008–E008.
